# Arabinogalactan-proteins of *Zostera marina* L. contain unique glycan structures and provide insight into adaption processes to saline environments

**DOI:** 10.1038/s41598-020-65135-5

**Published:** 2020-05-19

**Authors:** Lukas Pfeifer, Thomas Shafee, Kim L. Johnson, Antony Bacic, Birgit Classen

**Affiliations:** 1 Pharmaceutical Institute, Department of Pharmaceutical Biology, Christian-Albrechts-University of Kiel, Gutenbergstr. 76, 24118 Kiel, Germany; 2 La Trobe Institute for Agriculture & Food, Department of Animal, Plant and Soil Sciences, La Trobe University, Melbourne, Victoria 3086 Australia; 3 Sino-Australia Plant Cell Wall Research Centre, School of Forestry and Biotechnology, Zhejiang A&F University, Hangzhou, China

**Keywords:** Cell wall, Salt, Polysaccharides, Glycobiology

## Abstract

Seagrasses evolved from monocotyledonous land plants that returned to the marine habitat. This transition was accomplished by substantial changes in cell wall composition, revealing habitat-driven adaption to the new environment. Whether arabinogalactan-proteins (AGPs), important signalling molecules of land plants, are present in seagrass cell walls is of evolutionary and plant development interest. AGPs of *Zostera marina* L. were isolated and structurally characterised by analytical and bioinformatics methods as well as by ELISA with different anti-AGP antibodies. Calcium-binding capacity of AGPs was studied by isothermal titration calorimetry (ITC) and microscopy. Bioinformatic searches of the *Z. marina* proteome identified 9 classical AGPs and a large number of chimeric AGPs. The glycan structures exhibit unique features, including a high degree of branching and an unusually high content of terminating 4-O-methyl-glucuronic acid (4-OMe GlcA) residues. Although the common backbone structure of land plant AGPs is conserved in *Z. marina*, the terminating residues are distinct with high amounts of uronic acids. These differences likely result from the glycan-active enzymes (glycosyltransferases and methyltransferases) and are essential for calcium-binding properties. The role of this polyanionic surface is discussed with regard to adaption to the marine environment.

## Introduction

Seagrasses evolved from early monocotyledonous land plants, returning to the sea around 140 million years ago. Today, these marine angiosperms are a polyphyletic group containing about 60 species in four families (*Zosteraceae*, *Hydrocharitaceae*, *Posidoniaceae* and *Cymodoceaceae*). Seagrasses form important coastal ecosystems worldwide and support marine species, providing food and habitat. Human activities pose substantial risks for these sea meadows, prompting the need for protection and understanding of these valuable resources.

During adaption to the marine environment, several genes/gene families have been either lost (e.g. stomatal genes) or reduced (e.g. genes involved in the synthesis of terpenoids) whereas others have been regained (e.g. genes involved in sulfation of polysaccharides^1^). In contrast to other marine organisms, the cell wall of seagrasses is poorly understood. Beside ancestral traits of land plants, one would anticipate a habitat-driven adaption process to the new environment, which is characterised by multiple stressors (high amounts of salt, different seagrass grazers and bacterial colonization).

Genome sequencing of two *Zostera* species revealed that return to the marine habitat was accomplished by dramatic changes in cell wall composition^1,2^. Besides polysaccharides known from angiosperm land plants, the cell walls of seagrasses are characterised by sulfated polysaccharides, a common attribute of the macroalgae. For example, a sulfated D-galactan composed of the regular tetrasaccharide repeating unit [3-β-D-Gal-2(OSO_3_)-(1,4)-α-D-Gal-(1,4)-α-D-Gal-(1,3)-β-D-Gal-4(OSO_3_)−1,] was characterised from *Ruppia maritima*^3^. This ability to synthesize sulfated polysaccharides was lost during adaption to terrestrial as well as freshwater habitats and later independently regained by marine angiosperms. Another unique feature of cell walls of seagrasses is the occurrence of unusual polyanionic, low-methyl esterified pectins rich in apiose (Api*f*). In *Z. marina*, this characteristic polysaccharide is called zosterin and has a backbone of α−1,4-D-galacturonic acid (GalA*p*), substituted by 1,2-linked Api*f* oligosaccharides or single Api*f* residues^4,5^. Thus, cell walls of seagrasses are characterised by new combinations of structural polysaccharides known from both marine macroalgae and angiosperm land plants. In *Z. marina*, zosterin contributes to the polyanionic character of the cell wall matrix and possibly has crucial roles in osmotic adjustment to salt stress.

In addition to polysaccharides, glycoproteins are particularly important components of primary cell walls of land plants which can exhibit signalling functions during cell expansion and development. The highly glycosylated AGPs have gained particular interest due to their involvement in both wall architecture and cellular regulatory processes. Members of the hydroxyproline-rich glycoprotein family (classified in^6^), AGPs are ubiquitous in seed land plants^7^ and have also been found in ferns, lycophytes and mosses^8^. They are structurally characterised by large carbohydrate moieties comprised of arabinogalactans (AGs, normally >90% of the molecule) which are covalently linked via hydroxyproline (Hyp) to relatively small protein/peptide backbones (normally around 1–10% of the molecule). Distinct glycan modifications have been identified in different species and tissues and are suggested to influence both their physical properties and function. AGPs are therefore a good model to study cell wall adaptations to different environments. The AGs of seed plants mainly consist of type II (3,6)-galactans with 3-, 6- and 3,6-linked β-D-galactose (Gal*p*) residues, substituted with α-L-arabinose (Ara*f*) and often minor amounts of glucuronic acid (GlcA*p*) residues^7^. AGPs have been implicated in different developmental processes like cell growth, cell proliferation, pattern formation and reproduction^9–12^ and are sometimes also important in plant-microbe interactions^13^. Interestingly, massive upregulation of AGPs has been observed in salt-stressed tobacco cells, suggesting a role of AGPs in osmoregulation^14^.

To date, the presence of AGPs has not been established in any seagrass species. Therefore, we collected *Z. marina*, which is one of the ecologically most important macrophytes of the Baltic Sea, to search for AGPs. Investigation of the proteome of *Z. marina* L. identified sequences predicted to encode the highly glycosylated classical AGPs as well as low to moderately glycosylated chimeric AGPs. We utilized the specific interaction of AGPs with the dye β-glucosyl Yariv reagent (βGlcY) to isolate these glycoproteins and detect them by light microscopy. We established their major structural characteristics by different analytical methods as well as by interaction with different anti-AGP monoclonal antibodies. The AGPs from *Z. marina* exhibit special features not known for AGPs from land plants suggestive of a marine environment specialisation, which sheds further light on cell wall evolution, especially with regard to adaption to the marine habitat.

## Results

### Yield and composition of AGPs from *Z. marina*

High molecular weight fractions (HMFs) were isolated from the whole plant and from the different organs (roots, rhizome and leaves) by ethanol-precipitation of the supernatants prepared after centrifugation of the aqueous extracts. The HMF extracts had essentially similar monosaccharide compositions characterised by Gal- and Ara-rich polysaccharides and glycoproteins, except for in the root that had a lower content of Ara (Supplementary Table S1).

Precipitation of AGPs with βGlcY was undertaken from HMFs of whole plant and organs. The resulting AGP yields differed significantly. While the whole plant contained approximately 0.096% (m m^−1^ dry weight) AGPs, the rhizome and root AGP contents were much higher (0.144% (m m^−1^) and 0.138% (m m^−1^), respectively, whereas leaves contained very low AGP levels (0.005% (m m^−1^)).

Organic nitrogen quantification of βGlcY-precipitable AGPs from whole plant revealed 2.03% nitrogen, which corresponds to an approximate protein content (× 6.25) of 12.69%^15^. Colorimetric determination of Hyp detected 0.39% (m m^−1^) in the AGP from the whole plant sample, which equates to a Hyp content in the protein moiety of approximately 3.1% (w/w).

The AGPs from the different organs had Gal and Ara as their major monosaccharides but showed differences in their total Gal + Ara content (78–86%) as well as their Ara: Gal ratio (Table 1). Rhamnose (Rha*p*) was noticeably lower in the leaf AGP.

**Table 1:** Neutral monosaccharide composition (mol %) of the uronic acid reduced AGPs from the different *Z. marina* organs and from partial hydrolyses of whole plant AGP.

Monosaccharide	AGP_UR whole plant	AGP_URleaves	AGP_URrhizome	AGP_URroots	AGP_AH	AGP_Ox	AGP_UR+Ox
Gal	47.2	50.8	43.1	49.2	47.7	84.0	78.1
Ara	36.0	34.9	34.5	30.8	40.6	3.7	3.4
Rha	4.0	1.4	6.8	7.2	4.5	3.5	3.4
Man	2.5	2.6	3.3	2.0	3.3	5.0	1.3
4-OMe Glc	2.6	1.8	0.8	2.0	n.d.	n.d.	3.0
Glc	6.9	6.9	11.5	8.8	—	2.9	10.8
Xyl	0.8	1.6	—	—	3.9	0.9	—
Ara:Gal	1: 1.3	1: 1.5	1: 1.2	1: 1.6	1: 1.2	1: 22.7	1: 22.6

n.d., not detectable; AH, alkaline hydrolysis; Ox, oxalic acid hydrolysed; UR, uronic acid reduced.

Compared with the neutral monosaccharide composition prior to carboxyl reduction (Supplementary Table S2), a noticable increase in Glc content originating from GlcA was detectable in all AGP samples after uronic acid reduction (Table 1). The mass spectra of the peaks of Glc clearly revealed the presence of the characteristic fragments of a di-deuterated C6 (reduction of the carboxyl group with NaBD_4_). Furthermore, in all chromatograms of uronic acid reduced (UR) samples a peak appeared, which was not detected in the non-reduced AGPs. This peak with the relative retention time of 0.862 (relative to *myo*-inositol-hexaacetate) was clearly identified by its mass spectrum as C6 di-deuterated 4-O-methyl-Glc*p* (Supplementary Fig. S1).

Different partial hydrolyses with the whole plant AGP fractions were performed to gain insights into structural details of *Zostera* AGPs (Table 1). Through alkaline hydrolysis (AH) the protein backbone is removed whereas the carbohydrate composition remained largely unchanged. After AH, no Glc*p* was detected, which could be a consequence of the removal of trace levels of βGlcY under alkaline conditions. Mild acid hydrolysis (Ox) led to loss of most Ara*f* residues while reduction of uronic acids revealed the presence of both di-deuterated 4-OMe Glc*p* and Glc*p* originating from GlcA*p*.

### Structure of AGPs from *Z. marina*

AGP containing samples were subjected to linkage analysis by methylation after reduction of carboxyl groups and also after additional partial acid hydrolysis (Ox). For AGP after reduction of carboxyl groups, the molar recovery of branched residues (1,3,6-linked Gal*p*) was approximately equal to the molar recovery of non-reducing terminal residues (Ara*f*, Glc*p* and Rha*p*), indicating complete methylation. The analysis (Table 2) of the uronic acid reduced AGPs from whole plant revealed the different Gal*p* units typical for type II AGs with an extraordinarily high content of 1,3,6-linked Gal*p*, which indicates a highly branched structure, which was confirmed by size exclusion chromatography (SEC) (see below). The Ara*f* is mainly terminal and located in sidechains of the molecule. Interestingly, no 1,5-linked Ara*f* typical for many land plant AGPs was detected. Small amounts of 1,6-linked Man*p* might be part of N-glycans present on chimeric AGPs^16^ and have therefore not been included in the proposed structure (see below, Fig. 2). Mild acid hydrolysis of the sample prior to methylation led to near complete loss of Ara*f* residues and to an increase of 1,6-linked Gal*p*, indicating that Ara*f* is bound to Gal at C-3 of 1,6-linked Gal*p* branches. Deuterium-labelled Glc*p* was present as both terminal and 1,4-GlcA*p*. The primary fragments 207 (for deuterated terminal Glc*p*) and 235 (for deuterated 1,4-linked Glc*p*) were accompanied by no or only trace levels of the corresponding non-deuterated fragments (205 and 233, respectively), indicating that only GlcA*p* and not Glc*p* is present in the native AGP.

**Table 2:** Linkage analysis (mol %) of *Z. marina* AGPs before and after partial acid hydrolysis.

Monosaccharide	Deduced linkage type	*Z. marina* UR	*Z. marina* UR + Ox
Gal*p*	1,3,6-	40.0	32.3
	1,6-	1.7	39.1
	1,3-	7.4	10.7
	ter	—	1.9
Ara*f*	1,3-	4.2	1.0
	1,2-	1.3	—
	ter	23.7	1.7
Glcp*	1,4-	5.1	1.6
	ter	10.5	9.1
Man*p*	1,6-	3.0	1.0
Rha*p*	ter	3.1	1.6

*Derived from GlcA*p* by UR.

Ox, oxalic acid hydrolysed; UR, uronic acid reduced; ter, non-reducing terminal residues.

### Determination of molecular weight by size-exclusion chromatography

Absolute molecular weights of *Z. marina* AGPs and degraded products were determined by SEC separation and multi-angle light scattering (MALS) detection and hydrodynamic volumes were calculated using commercially available pullulan standards (Supplementary Table S3). The absolute molecular masses determined by MALS were in a range typical for AGPs and always much higher compared to their hydrodynamic volumes. This showed a highly branched structure of all the AGP molecules (explaining a large mass in a small volume). Partly, these differences could be caused by the different structures of pullulan standards, which are linear and unbranched polymers^17^, compared with branched AGPs. As expected, chemical modifications by either reduction of uronic acids or oxalic hydrolysis (Ox) decreased the absolute molecular masses and hydrodynamic volumes, due to loss of hydration (UR^18^) and loss of mainly Ara*f* by treatment with Ox. In all chromatograms some higher order aggregates resulting from self association were present; whether or not this reflects an *in vivo* property or is an artefact of the fractionation procedure cannot be distinguished. It is not unusual to see such aggregation unless the chromatography is conducted under strong dissociating conditions, such as chaotropic reagents (eg urea/guanidine hydrochloride) but this was not compatible with the SEC-MALS detection^19^.

### Binding of *Z. marina* AGPs to antibodies raised against land plant AGPs

**Figure 1:**
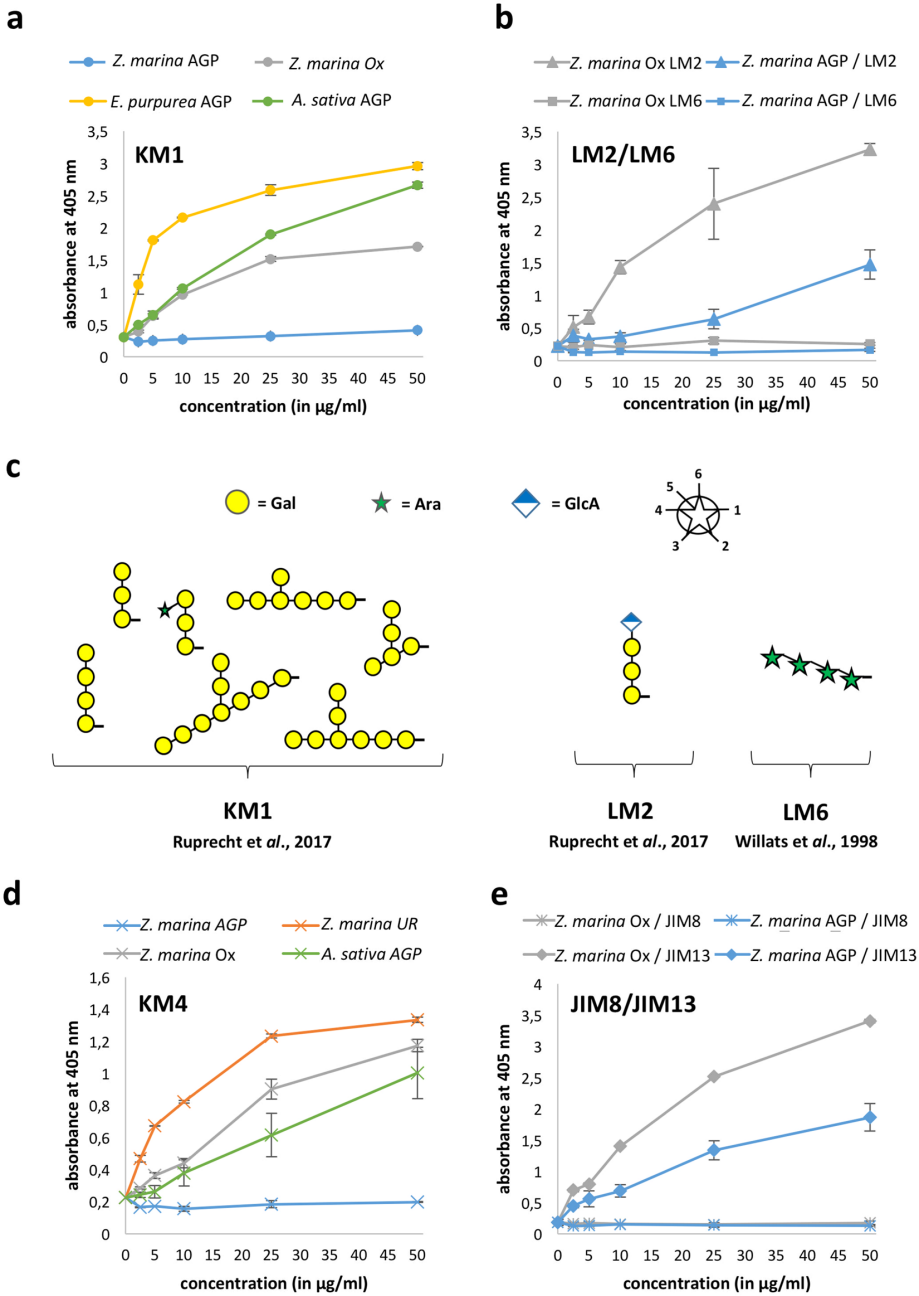
Reactivity of *Zostera marina* AGPs and their partial hydrolyses (with oxalic acid = Ox; by reduction of uronic acids = UR) with antibodies directed against AGP glycans by ELISA. (**a**) KM1 antibody with *E. purpurea* AGP and *A. sativa* AGP as positive controls. (**b**) LM2 and LM6 antibodies. (**c**) oligosaccharides with strong binding affinity to KM1, LM2 and LM6, respectively^22^. (**d**) KM4 antibody with *A. sativa* AGP as positive control. (**e**) JIM8 and JIM 13 antibodies.

The native *Z. marina* AGPs and their partially degraded products were investigated for their ability to bind to the antibodies LM2, LM6, JIM8, JIM13^20–23^, KM1 (raised against *Echinacea* AGP^24^), and KM4 (raised against *Avena* AGP^25^) (Fig. 1). With KM1, the native AGP from *Z. marina* showed no reactivity. After mild acid hydrolysis good binding was evident (Fig. 1a), due to increase in 1,6-linked Gal*p* residues (Table 2). These 1,6-linked Gal*p* residues are present in the original antigen (*Echinacea* AGP^24^) and an AGP from the terrestrial monocotyledonous plant *Avena sativa*^26^ and known to be part of the binding epitope (Fig. 1c^22^). The epitope of LM2 comprises some 1,6-linked Gal*p* residues with a terminal GlcA*p* (Fig. 1c^22^). The native *Z. marina* AGPs show moderate binding which increases after mild acid hydrolysis (Fig. 1b), known to increase 1,6-linked Gal*p* residues. LM6, which is directed against a 1,5-linked Ara*f* chain (Fig. 1c), does not bind to *Z. marina* AGPs, thus confirming that this linkage is absent in this seagrass AGP (Fig. 1b, see also Table 2). KM4, which was raised against an AGP from *Avena sativa* which does not contain uronic acids^26^, showed no binding to the native, strongly charged *Z. marina* AGPs. After mild acid hydrolysis and especially after reduction of uronic acids, binding exceeded that of the original antigen (Fig. 1d). JIM8 and JIM13 are antibodies directed against AG glycans on AGPs but the precise epitope is not defined. Whereas JIM13 reacted with *Z. marina* AGPs, especially after mild acid hydrolysis, there was no interaction with JIM8 (Fig. 1e).

**Figure 2:**
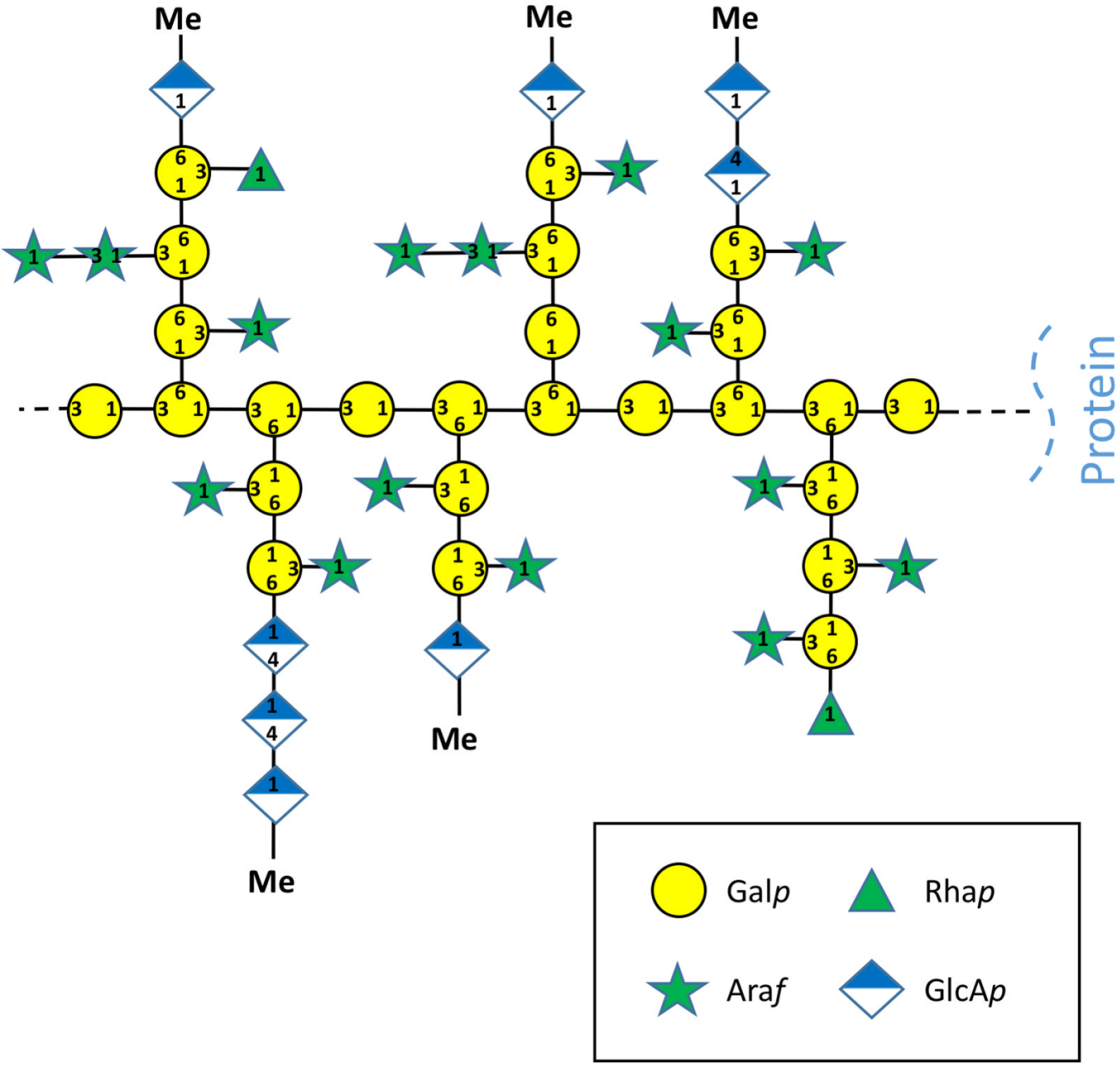
Structural proposal for the polysaccharide moiety of *Zostera marina* AGP based on molar portions detected in linkage analysis (see Table 2).

Combining the structural data of the polysaccharide moiety and antibody epitope binding studies, a predicted *Z. marina* AGP glycan structure is proposed (Fig. 2). This model incorporates both known structural features characteristic of angiosperm AGPs and features unique to *Z. marina* such as a very high degree of branching, consistent with the high amounts of 1,3,6-linked Gal*p* residues and only traces of 1,6-linked Gal*p*- containing side chains, and high amounts of terminal oligosaccharides rich in uronic acids, especially 1,4-linked GlcA*p* residues and terminal 4-OMe GlcA*p*. For comparison with *Z. marina* AGP an *A. sativa* AGP structure was added (see Supplementary Fig. S2).

### Calcium-binding experiments by ITC

**Figure 3:**
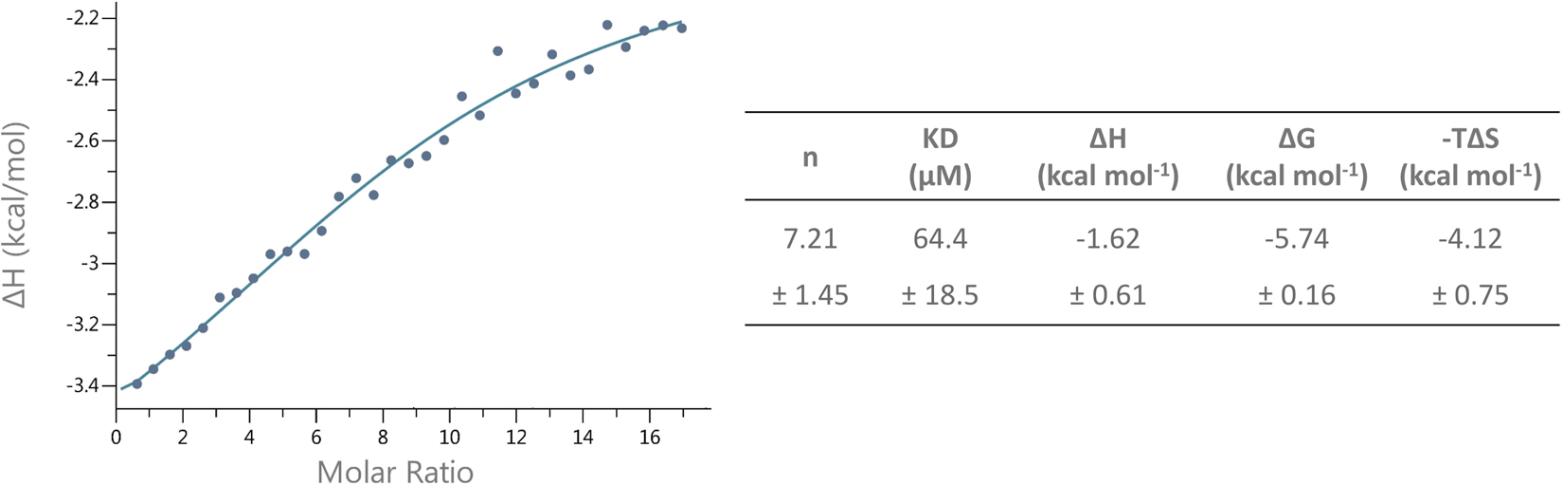
Isothermal titration calorimetric detection of calcium-binding to *Zostera* AGP. Thermodynamic values of calcium-binding assay using ITC. Fitting curve using the one-site binding model. The values in the table were averaged from three measurements.

ITC showed the calcium binding capacity of *Z. marina* AGP and gave information about its thermodynamic binding parameters (Fig. 3). One important value for binding studies is the dissociation constant (K_D_), which is in the micromolar range. The reaction is characterized as exergonic (negative ΔG), exothermic (negative ΔH) and mainly entropy-driven (high –TΔS), consistent with the second law of the thermodynamics (Fig. 3). As a control experiment, AGP from fruits of *A. sativa*, which is free of uronic acids^26^ (for structural proposal see Supplementary Fig. S2), was used in the same test system and showed no binding to Ca^2+^ in the ITC thermogram.

### Detection of AGPs and calcium-binding in tissues by light microscopy

**Figure 4:**
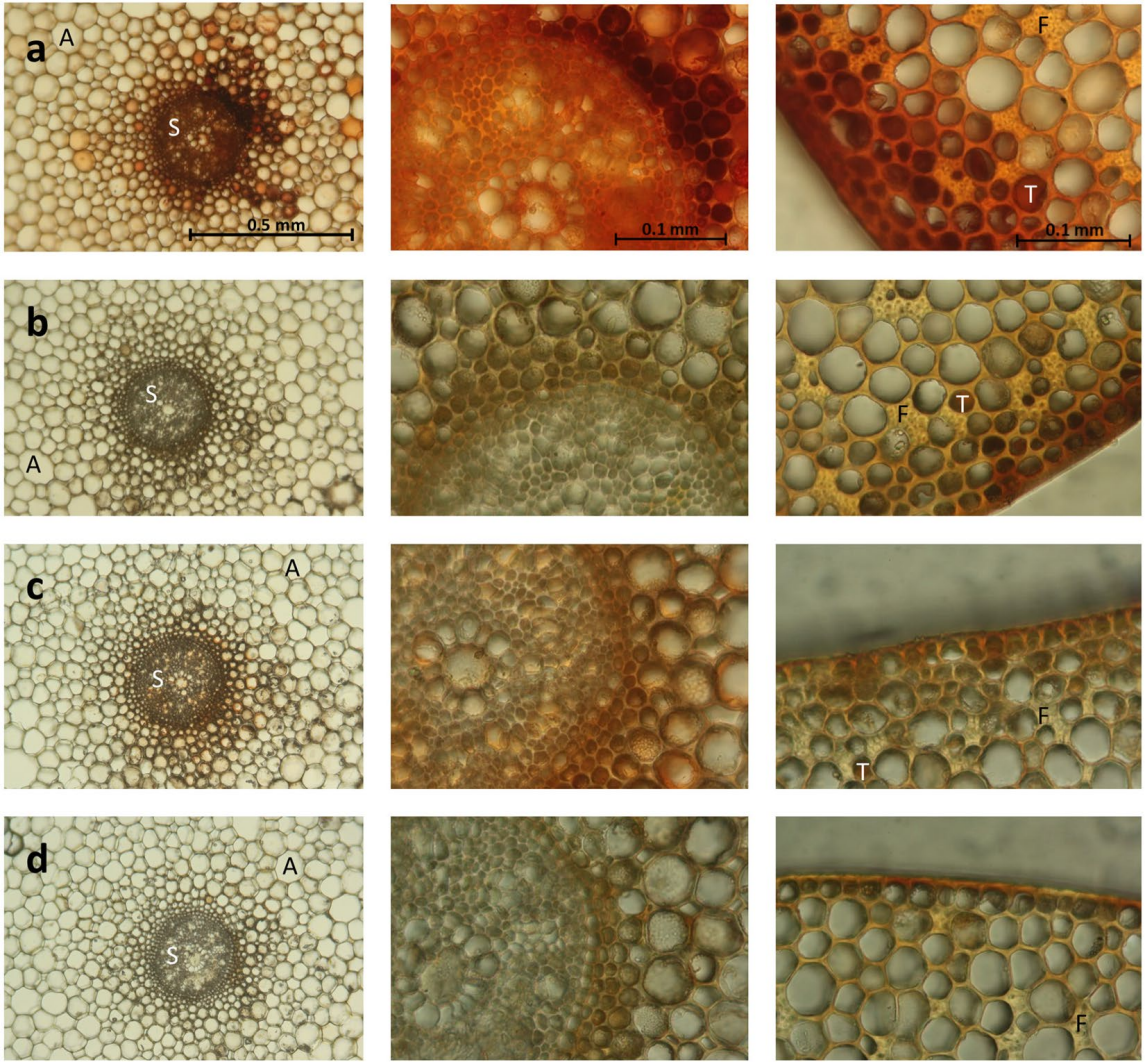
Co-localisation of calcium binding and occurrence of AGPs in *Zostera marina* rhizome detected by microscopy. (**a**) Red staining of AGPs by βGlcY in rhizome cross sections. (**b**) Negative control without βGlcY staining. (**c)** Rhizome cross section incubated in calcium chloride solution and stained with Alizarin S. (**d**) Negative control incubated in water and stained with Alizarin S. A = air lacunae, S = stele, F = vascular fibre bundles and T = tannin cells (according to^27,28^).

Cross sections of fresh rhizomes (Fig. 4a–d) showed the general anatomy evident for land plant rhizomes: epidermis, cortex and a central cylinder (stele). Furthermore, anatomical features described for other seagrass rhizomes^27,28^ were detected. In the outer part of the parenchymatous cortex, tannin-containing cells as well as large fiber bundles are noticeable. Other typical elements are several air lacunae present in the cortex. Two opposite cortical vascular bundles, which have also been detected in rhizomes of *Zostera caulescens*^28^ are also present (not shown). The red-coloured βGlcY was used to stain AGPs (Fig. 4a). AGPs were mainly detected in the stele and the surrounding inner layers of the cortex, the two cortical vascular bundles (not shown) as well as in the outer layers of the cortex. Staining with Alizarin S (Fig. 4c), which is an anthraquinone dye used to detect calcium deposits, revealed co-localization of calcium and AGPs.

### Identification of AGP protein sequences in *Z. marina*

**Figure 5:**
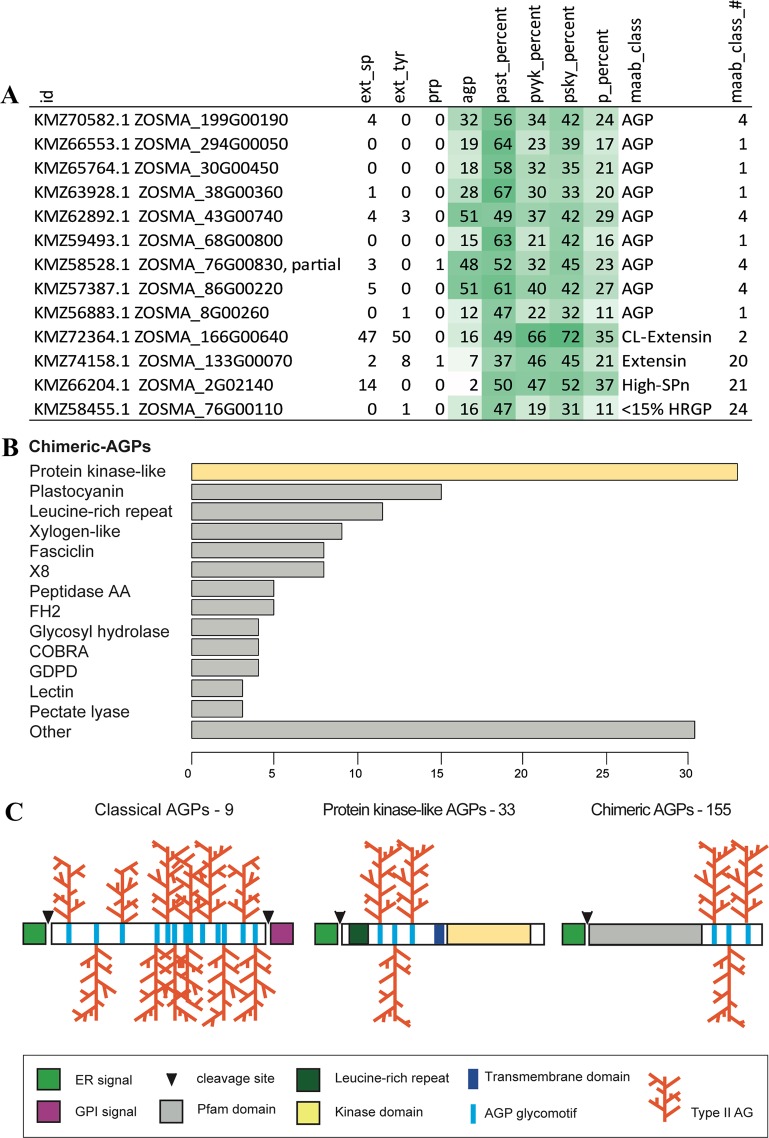
HRGP and AGP classes identified in the *Zostera marina* proteome. (**A**) The majority of HRGPs identified by the MAAB pipeline are classical AGPs of Class 1: GPI-AGPs and Class 4: non- GPI-AGPs. The number of glycomotifs characteristic of extensins (ext_sp and ext_tyr), PRPs and AGPs in a given sequence is shown. The percentage amino acid bias characteristic of AGPs (PAST), PRPs (PVYK) and extensins (PSKY) is shaded in light green (0–25%), medium green (25–50%) and dark green (50–75%). (**B**) Chimeric AGPs, identified by first screening the genome for sequences that include a region with at least 3 AG glycomotifs and a Pfam domain (ragp package, GitHub). The major chimeric AGP classes with 3 or more representatives are shown with similar Pfam families grouped by clan. A full list of Pfam families and most common domain architectures are shown in Figs. S4 and S5. (**C**) Schematic of predicted AGP structures of classical AGPs and chimeric AGPs that include protein-kinase like AGPs and other chimeric AGPs in *Zostera marina*.

The *Z. marina* genome contains 38,875 coding sequences (CDSs)^1^. Hydroxyproline-Rich Glycoproteins (HRGPs) and in particular, classical AGPs, were identified using the established motif and amino acid bias ‘MAAB’ pipeline^29,30^. Chimeric AGPs were identified by first screening the proteome for sequences with predicted AG regions, followed by filtering for sequences containing at least one detectable protein domain (Pfam), and a signal peptide (SignalP 5.0). MAAB identified 13 sequences that are recognized as HRGPs (Fig. 5, Supplementary Fig. S3). The majority of these sequences are classical AGPs, 5 of which contain a GPI-anchor signal sequence (MAAB Class 1) and 4 non- GPI-AGPs (MAAB Class 4) (Fig. 5A). Additionally, there is one classical extensin (MAAB Class 2), two sequences with shared bias (MAAB Class 20 and 21), that contain both extensin and AGP glycomotifs, and a sequence from the <15% HRGPs (MAAB Class 24) (Fig. 5A). These proportions are similar to those found in the genomes of terrestrial monocots^29,30^.

In addition to the classical AGPs, there are a further 188 chimeric AGPs (33 protein kinase-like AGPs + 155 other chimeric AGPs) that contain one or more protein domains as identified in Pfam, a signal peptide and at least one AG-motif rich region (Fig. 5B). The most common chimeric AGPs observed are the protein kinase-like AGPs (PKGPs), copper oxidase plastocyanin/ENOD-like AGPs (PAGs), lipid-transfer xylogen-like AGPs (XYLPs) and fasciclin-like AGPs (FLAs) (Fig. 5B). The amount of glycosylation is predicted to vary between the highly glycosylated classical AGPs that contain AG glycomotifs throughout the protein backbone and the chimeric AGPs that are likely to be low to moderately glycosylated in different regions of the protein backbone (Fig. 5C). The chimeric AGPs also show high variation in domain organization, i.e. where the Pfam is located in relation to the AG region and how many of each, even within the same group (Supplementary Figs. S4 and S5).

The *Z. marina* AGP protein sequences are largely characteristic of known angiosperm AGP protein backbones suggesting the structural differences in glycan structures likely result from the glycan-active enzymes (glycosyltransferases (GTs) and methyltransferases) acting on the *Z. marina* AGPs. As a consequence the complement of GT and methyltransferase families implicated in AG glycan assembly were interrogated in the *Z. marina* genome.

### Glycan-active enzyme complement of *Z. marina*

The glycan structures of AGPs are primarily assembled by GT enzymes from the GT14, GT31, GT61 and GT77 families and methyltransferases from the DUF579 family^31–34^. *Arabidopsis thaliana* sequences were used to search the *Z. marina* genome and identified 25 GT31s (galactosyltransferases (GalTs) and N-acetylgalactosaminyltransferases (GalNAcTs), slightly higher than the number observed in other viridiplantae^31^. GT31 members are proposed to add the first Gal to Hyp as well as build the β−1,3 Gal backbone and β−1,6 Gal side-chains. When compared to the *A. thaliana* homologs (the only organism for which some GT31 functions have been assigned) there is a minor expansion in Hyp GalTs (Clade 7-V) which initiate glycosylation of the AGP protein backbone, and additional expansion of Clade 10-IV of unknown function (Supplementary Fig. S6). *Z. marina* has 14 GT14s, again, in line with the 10–18 found in other viridiplantae, with minor expansions in expansions in clade B5 (glucuronosyltransferases (GlcATs) and clade B2 of unknown function (Supplementary Fig. S7). The GT77 family also contains a number of well-characterised arabinosyltransferases (AraTs) and arabinofuranosyltransferases (Ara*f*Ts)^35,36^. Compared to *A. thaliana*, most clades contain similar numbers of homologs with clades D and E the most likely candidates for containing the relevant AGP-active 1,3-Ara*f*Ts, whereas the rhamnogalacturonan-II-active enzymes (clade B) are reduced (Supplementary Fig. S8).

**Figure 6:**
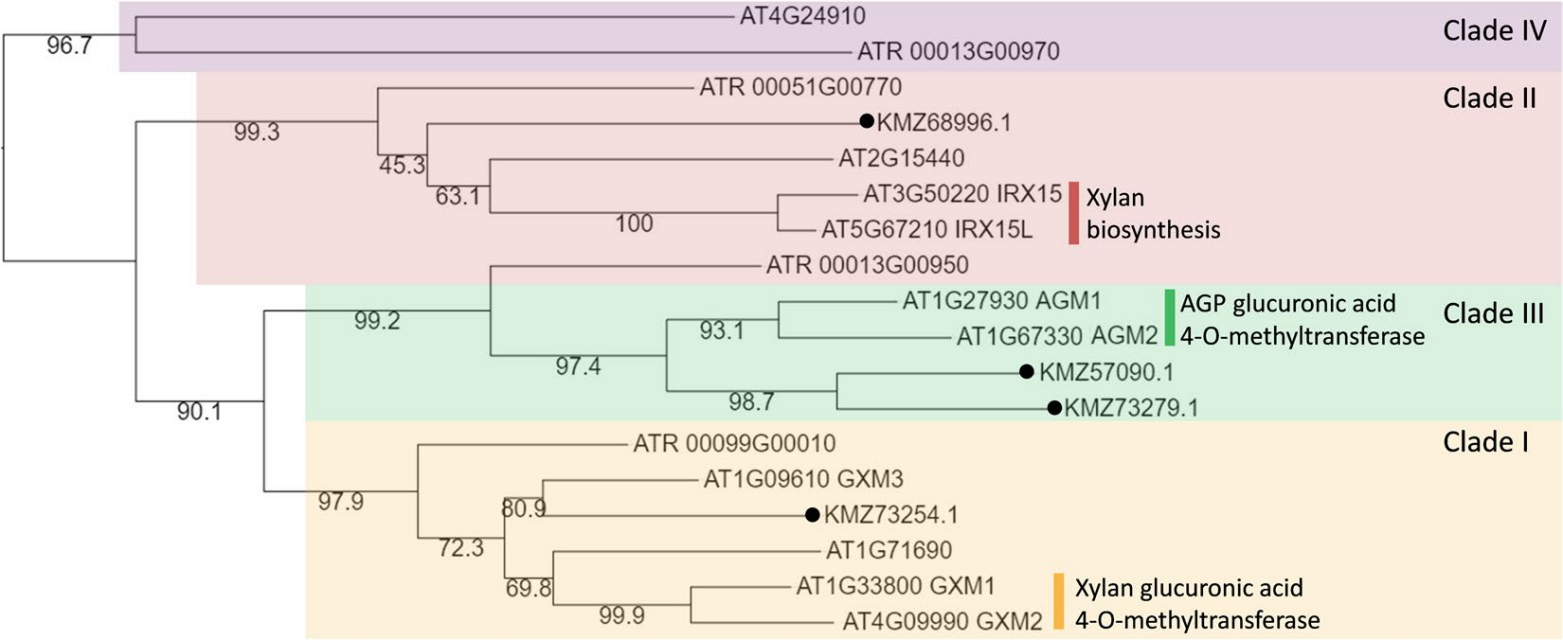
Phylogeny of DUF579 family members from *Zostera marina* (KM accession numbers, highlighted with black circles), and *Arabidopsis thaliana* (AT). Clades labelled as in^34^. Genes with known function indicated. For equivalent phylogenies of GT families, see Supplementary Figs. S6–S8.

Conversely, the GT61 and DUF579 families assessed had gene complements significantly differing from those found in other vascular plants. *Z. marina* has fewer GT61s of the A, D and F clades (Supplementary Fig. S8), thought to contain the relevant 1,5-AraTs, compared to the 3–10 usually found in vascular plants (and 20–50 in the Commelinidae land monocots)^33^. Similarly, there are fewer members of the DUF579 methyltransferase family (Fig. 6). Within this family, Clade III, has been shown to exhibit methylation of AGP glycans in *A. thaliana*^34^. *Z. marina* contains two sequences in this clade: KMZ57090.1 and KMZ73279.1 which we propose are the most likely candidates responsible for the extensive methylation of the AGP terminal GlcA*p* residues.

## Discussion

*Z. marina* is of major ecological importance^37^ and the biomass is used as environmentally friendly and non-flammable insulating material, properties that are influenced by the composition of their cell walls. Despite this, knowledge of the composition of the cell walls within the polyphyletic group of seagrasses is largely restricted to the major polysaccharides that are unique to marine plant and algal species, and include an apiogalacturonan (“zosterin”)^4,5,38^ and sulfated galactans^3,39,40^. Whether seagrass cell walls/cell surfaces also contain a similar complement of minor polysaccharides/glycoproteins, particularly those implicated as signalling molecules in terrestrial plants, such as the AGPs, was of particular interest from both an evolutionary and plant development perspective.

AGPs are key constituents of the extracellular matrix of flowering land plants with important functions in processes such as cell growth, cell proliferation, pattern formation, reproduction and plant-microbe interactions^7,9–12,41^. Interestingly, accumulation of AGPs has been observed in the media of salt-stressed suspension cultures of tobacco, tomato, *Acacia*, *A. thaliana*^14,41,42^ and *Dactylis*^43^, supporting a role of AGPs in osmoregulation. In *Brassica*, a significant accumulation of AGPs in the xylem sap after 24 h of salt treatment was observed^44^. Studies on *Medicago sativa* leaves showed an increase of AGPs recognized by the JIM8 antibody in NaCl-treated plants^45^. When seagrasses evolved from monocotyledonous land plants and colonized the coastal marine ecosystems this was possibly the most severe change of habitat in evolution of flowering plants, mainly due to salt stress. Salinity impairs plant growth and development *via* both a water stress due to osmotic pressure and thus the reduction in water availability and also via a direct cytotoxicity due to excessive uptake of ions, mainly sodium (Na^+^) and chloride (Cl^−^) and a critical loss of potassium (K^+^). The proposed role of AGPs in salt stress made them an ideal candidate to investigate how changes in wall components could play a role during salt adaption to highly saline conditions.

This study represents the first isolation and characterisation of AGPs in seagrasses. Interestingly, the amount of water-soluble AGPs in roots and rhizomes of *Z. marina* was around 20 fold higher than in leaves. One reason for the higher content of AGPs in rhizomes and roots might be due to the fact that leaves persist for only one year whereas rhizomes and roots are perennial. If one function of AGPs is protection against salt, the longer lasting organs may have a higher AGP requirement. In the marine angiosperm *Ruppia* sulfated galactans possibly involved in salt protection are also mainly present in the rhizomes^3^.

The isolated AGP-fraction from *Z. marina* fulfills the general criteria which are typical for many AGPs like a large AG moiety that consists mainly of 3-, and 3,6-linked β-D-Gal*p* residues, substituted primarily with terminal α-L-Ara*f* residues, a small protein part mostly rich in Hyp and the ability to be precipitated with Yariv phenylglycosides, for example, the βGlcY reagent. Molecular weight, protein content and amount of Hyp in *Zostera* are in a range reported for land plant AGPs. Similarly, the complement of genes in the draft *Z. marina* genome expected to encode proteins that would be precipitated by βGlcY is broadly in line with other vascular plants. Nine classical AGPs were identified, broadly similar to those observed in the commelinid monocots^30^. The chimeric AGPs (that contain one or more Pfam domains) are considerably more numerous than the classical AGPs and include the most common chimeric classes, FLAs, PAGs and XYLPs and surprisingly, a large number of PK-like AGPs. Interestingly, PK-like AGPs were identified in the marine brown alga *Ectocarpus* and proposed to have roles as sensor molecules^46^.

Two main structural features were identified in *Z. marina* AGPs that are distinct from angiosperm land plant AGPs. Firstly, high branching combined with a high Ara*f* content was observed, especially in AGPs isolated from the rhizome. For *A. thaliana*, it has been shown that Ara*f* biosynthesis is important for salt stress tolerance. Mutations in a gene which is required for UDP-Ara biosynthesis led to reduced root elongation under high salinity and could be healed by application of either exogenous Ara or gum arabic, a commercially available gum from *Acacia senegal* rich in AGPs^47^. A survey investigating different resurrection plants from South Africa revealed that Ara-rich polymers plasticize resurrection plant cell walls in case of desiccation and facilitate rehydration^48^.

An even more striking feature of *Z. marina* AGPs are high amounts of GlcAps, especially 1,4-linked GlcA*p* and terminal 4-OMe GlcA*p* residues. The presence of significant levels of the 4-O-Me-GlcA*p* is not a common feature of land plant AGPs, but has been detected rarely, e.g in AGPs from *Raphanus*^49,50^. The significant differences in content and composition of AGPs in *Z. marina* suggest specific functional adaptations to the marine environment. Given the unusual AG glycan structures, the genome was searched for key enzyme families known to be involved in AG synthesis. The genome contained a relatively standard set of sequences from the GT31 family (3- and 6- GalTs), GT77 (AraTs) and GT14 family (GlcATs), with members present from all the major clades characterised in each family. Conversely the GT61 (AraTs) and DUF579 (methyltransferases) families have markedly divergent distributions of members. This corresponds to the lack of 1,5-linked Ara*f* residues (fewer GT61) and extensive methylation (DUF579) of GlcA*p* residues in *Z. marina*. The enzymes responsible for methylation of GlcA*p* in AGPs have recently been identified in *A. thaliana*^34,51^. Two sequences were identified as the most likely candidates responsible for the extensive methylation of the AGP terminal GlcA*p* residues in *Z. marina*. Since no gene family expansion was observed in DUF579 family Clade III, the markedly increased methylation observed in *Z. marina* AGPs is likely due to their unique upregulation (transcriptional or post-translational) in response to the exposure to a highly saline environment. In future, experimental verification of either altered transcript abundance or activity will further our understanding of this unique structural modification.

Uronic-acid rich residues are likely to lead to a polyanionic surface of *Z. marina* AGP which is likely of great importance with regard to adaption to the marine environment^52^. Important is the ability of uronic acids to bind Ca^2+^, and it has been shown, that uronic acid containing AGPs bind Ca^2+^ more strongly than does pectin^52^. The authors identified stoichiometric Ca^2+^ binding by paired GlcA carboxyls present in AGPs and this model is consistent with our findings for *Z. marina* AGPs. It has been proposed that subtle structural variations of the Ca^2+^-binding subunit of AGPs helps to discriminate effectively against binding of Na^+^^53,54^. One possibility for this fine-tuning of ion-binding affinity might be *O*-methylation of different monosaccharides; e.g. the occurrence of 3-OMe Rha*p* in moss AGPs^55–57^ and especially 4-OMe GlcA*p* in *Z. marina* AGP. This confirms the results of Lamport and Várnai^53^ for binding of different AGPs to Ca^2+^ in a micromolar range. It is worth noting that a different methodology was used by these authors. We used the Chelex resin for Ca^2+^ removal, which has high affinity to bivalent cations. Our control experiment with an uronic acid-free AGP from the monocotyledonous land plant *Avena sativa* (no binding to Ca^2+^) proved that uronic acid-rich terminal groups of *Z. marina* AGPs are essential for the Ca^2+^-binding properties. This finding is also supported by light microscopy of cross sections of the rhizomes, which revealed co-localisation of AGPs and Ca^2+^.

Ca^2+^ ions play a crucial role in both the regulation of transport and exclusion of Na^+^ and other mineral ions at the plasma membrane of plant cells, and for some time it was known that Ca^2+^ protects an extremely salt-sensitive species (*Phaseolus vulgaris*) against damage caused by NaCl present in the medium^58^. Investigations on cotton supported the finding that Ca^2+^ protects membranes from harmful effects of Na^+^ and maintained membrane integrity^59^. As AGPs can be linked to the plasma membrane by a GPI-anchor, binding of Ca^2+^ to AGPs might build a barrier protecting the plasma membrane against high Na^+^-concentrations in the marine environment.

Beside the possible barrier function of uronic-acid rich, Ca^2+^-binding AGPs close to the plasma membrane, another important mode of action of AGPs might be signalling. Signalling caused by AGPs might occur via either Ca^2+^-ions^54^, via oligosaccharides enzymatically cleaved from the AG glycan moieties of AGPs or by association of GPI-anchored AGPs with classical transmembrane proteins. It has been proposed, that Ca^2+^-release from AGPs is an auxin-dependent process via regulation of the plasma membrane (PM) H+ -ATPase, which generates a lower periplasmic pH leading to dissociation of AGP-Ca^2+^ carboxylates^52,54^. In *Torenia*, an AGP called AMOR has been identified which is responsible for cell-cell-communication in the pollen-pistil interaction. Interestingly, the terminal disaccharide 4-OMe GlcA*p*-β-(1 → 6)-Gal*p* is necessary and sufficient for activity^60^. The exact signalling pathways either by Ca^2+^ or by oligosaccharides have yet  to be elucidated for *Z. marina*.

## Conclusions

To date, the presence of AGPs has not been established in any seagrass species. We were able to isolate AGPs from *Z. marina* and structurally characterize these glycoproteins by analytical methods as well as by ELISA with different anti-AGP antibodies. The glycan structures exhibit unique features, including a high degree of branching and a high content of terminating 4-OMe GlcA*p* residues not known from land plants. These differences likely result from the glycan-active enzymes (GTs and methyltransferases). Bioinformatic investigation identified protein backbones belonging to 9 classical AGPs and a large number of chimeric AGPs. This group showed unusual dominance of PK-like AGPs, which are proposed to work as sensor molecules. For a deeper insight into structure-function correlation, the Ca^2+^-binding capacity of *Z. marina* AGPs was investigated by microscopy and ITC. In contrast to AGP from the land plant *A. sativa*, it showed binding in the range of the important Ca^2+^ binding partner calmodulin. These findings shed light on the adaption process from land to the marine habitat.

## Methods

### Materials

Fresh green, epiphyte-free samples of *Zostera marina* L. were collected after a stormy night at the beach near Olympic Centre Schilksee, Kiel (54°25′39.0″N 10°10′17.5″E) in March 2018. They were cleaned from coarse pollution, rinsed in tap water and freeze-dried. Half of the material was separated into the different plant organs (roots, rhizome and leaves) before freeze-drying.

### Isolation of high molecular weight fraction (HMF)

The dried plant material was milled in a MF 10 basic grinder (IKA-Werke GmbH & Co.KG, Staufen, Germany) with a sieve size of 1.0 mm. Ground material was then extracted with tenfold (w v^−1^) of 70% acetone (v v^−1^) for 22 h at 4 °C under constant movement on a shaker (Edmund Bühler GmbH, Bodelshausen, Germany). Acetone was removed by vacuum filtration. This procedure was repeated four times to ensure a successful removal of tannins and colored impurities. Afterwards an aqueous extraction (1:10 (w v^−1^)) of the air-dried plant material was carried out following the procedure of^10^. The resulting precipitate, the high molecular weight fraction (HMF), was separated by centrifugation at 19,000 *g*, 4 °C for 30 mins and freeze-dried. Samples of whole plant, rhizome, root and leaves were subjected to this same extraction procedure.

### Isolation of AGPs

For purification of AGPs, β-glucosyl-Yariv reagent (βGlcY) was used and isolation was done according to^61^.

### Analysis of neutral monosaccharides

Determination of the monosaccharide composition was carried out according to^62^. Either HMF (2–5 mg) or AGP (1–3 mg) fractions were mixed with 0.5 mg of the internal standard *myo-*inositol, then hydrolysed, reduced and acetylated. Identification and quantification of monosaccharides was performed with gas chromatography (GC) with parallel flame ionization detection (FID) and mass spectrometry (MS) (GC + FID: Agilent 7890B, Agilent Technologies, USA; MS: Agilent 5977B MSD, Agilent Technologies, USA; column: Optima-225, 25 m, 50 µm, 0.25 µm; helium flow rate: 1 ml min^−1^; temperature 230 °C; split ratio 30:1). A standard mixture was analysed, peak identification and quantification of known peaks was done through relative retention times and response factors, unknown peaks were identified by MS.

### Acid and alkaline partial hydrolyses of AGPs

As described in^63^ the samples were acid hydrolyzed in 12.5 mM aqueous oxalic acid (Ox) and heated under pressure for 5 h at 100 °C (Wheaton V-Vial; Bioblock Scientific, Thermolyne Corp., USA). Alkaline hydrolysis (AH) was performed as described in^64^.

### Reduction and labeling of uronic acids with NaBD_4_

AGP samples (40 mg) were solubilized in 40 ml of deionized water and treated with N-cyclohexyl-N’-(2-morpholinoethyl) carbodiimidemethyl-*p*-toluenesulfonate and solutions of NaBD_4_^65^. After this treatment the sample was purified through dialysis (MWCO 6–8 kDa) for 3 days at 4 °C against deionized water and freeze-dried.

### Linkage-type analysis

Freeze-dried AGP samples (2–8 mg) of was dissolved in dimethylsulphoxide (DMSO) and methylated according to^66^. The partially methylated alditol acetates were extracted with dichloromethane and separated via GC (instrument: Agilent 7890B, Agilent Technologies, USA; column: Optima-1701–0.25 µm, Machery & Nagel, Düren, Germany; helium flow rate: 1 ml min^−1^; temperature: initial 170 °C for 2 min, with rate 1 °C min^−1^ to 210 °C, then with rate 30 °C min^−1^ until 250 °C is reached and following hold time of 10 min) and analyzed by MS and FID as above.

### Determination of Hydroxyproline (Hyp) content

Hyp content was determined colorimetrically according to^67^.

### Elemental Analysis

Quantitative determination of nitrogen was performed with HEKAtech CHNS Analyzer (HEKAtech GmbH, Wegberg, Germany) through burning in an excess of oxygen. The resulting combustion products were further analyzed. To calibrate the system aminobenzenesulfonamide was used.

### Size-exclusion chromatography (SEC)

SEC analysis was performed as described in^68^. For calculation of hydrodynamic volumes, different pullulans with the masses 85.3 × 10^4^ Da, 38.0 × 10^4^ Da, 18.6 × 10^4^ Da, 2.37 × 10^4^ Da, 1,22 × 10^4^ Da (all from Shodex, Japan), 4.8 × 10^4^ Da and 10.0 × 10^4^ Da (both Honeywell Fluka, USA) were used. They were prepared and injected in the same way as the sample. A standard calibration curve was done and hydrodynamic volume of the samples were determined through elution time. For absolute molecular weight the peaks of MALS signal were integrated and results were fitted with a second order exponential function.

### Enzyme-linked immunosorbent assay (ELISA)

Binding of the different monoclonal AGP-antibodies JIM8, JIM13, LM2, LM6, KM1 and KM4 was investigated in an indirect ELISA. First the 96-well plates (Nunc, Nalge Nunc International, Roskilde, Denmark) were coated with different concentrations of either native AGP or partial hydrolysed AGP (0, 2.5, 5, 10. 25, 50 µg ml^−1^ in double-distilled water, 100 µl per well) at 37.5 °C with open cover for 3 days. Afterwards the plates were washed three times with phosphate buffered saline (PBS) buffer (pH 7.4 with 0.05% Tween 20) and blocked with 0.1% (wv^−1^) BSA in PBS (pH 7.4, 200 µl per well, 1 h at 37.5 °C). The blocked plates were washed again with the same washing buffer as described above. Next 100 µl of the different antibodies (KM1 and KM4 diluted 1:10 (vv^−1^), JIM8, JIM13, LM2 and LM6 diluted 1:20 (vv^−1^)) was pipetted in each well and the plates were incubated for 1 h at 37.5 °C followed by a three-times washing step. The secondary antibody (either Anti-Rat-IgG or Anti-Mouse-IgG conjugated with alkaline phosphatase, Sigma-Aldrich Chemie GmbH, Taufkirchen, Germany) was added in a dilution of 1:500 (vv^−1^) in PBS. After incubating and washing, color was developed by addition of 0.1 mg ml^−1^ *p*-nitro-phenylphosphate in 0.2 M TRIS buffer (100 µl well^−1^). Absorption was measured at 405 nm in an ELISA reader (Tecan Spectra Thermo). Samples were tested in duplicate.

### Light microscopy

To visualize AGPs in the rhizome, cross sections derived from handmicrotome (Euromex Microscopen BV, Arnhem, Netherlands) slicing of fresh rhizomes of *Z. marina* were stored in 80% (v v^−1^) ethanol. Afterwards slices were incubated in βGlcY solution (1 mg ml^−1^ in 0.15 M NaCl) for 24 h and washed afterwards three times with 0.15 M sodium chloride solution (negative control without βGlcY).

For detection of calcium-binding regions in the rhizome, sections were either incubated with 25 mM calcium chloride solution for 15 min or with destilled water as a negative control. Afterwards both samples were treated with 2% m v^−1^ Alizarin S reagent solution (pH 4) for 5 min. After washing three times with 80% (v v^−1^) ethanol, the sections were investigated by light microscopy (Carl Zeiss AG, Oberkochen, Germany, pictures acquired by Canon EOS 1000D, Canon AG, Tokyo, Japan).

### Calcium binding experiments by ITC

Binding of Ca^2+^ to *Z. marina* AGP was measured with isothermal titration calorimetry (ITC) on the instrument MicroCal PEAQ-ITC (Malvern Instruments Ltd., Malvern, UK). As a negative control Avena sativa AGP, free of uronic acids^26^ was used. 280 µl of AGP samples in a concentration of 20 µM were deposited in the sample cell and titrated with 40 µl of calcium chlorid solution in a concentration of 2 mM. The injection pipett was stirred with 750 rpm, one single injection of 0.2 µl was made at the beginning and 32 injections of 1 µl followed. Each with a spacing of 150 s. The results were fitted in the MicroCal PEAQ-ITC analysis software (Malvern Instruments Ltd., Malvern, UK) and the K_D_ value, as well as other thermodynamic values were calculated. To remove calcium contaminants in the AGPs before measurement, the samples were treated with Chelex sodium salt (Sigma-Aldrich Chemie GmbH) with a batch method and freeze-dried afterwards. Titrant and titrated solution were soluted in MES buffer solution (20 mM, pH 6) to avoid calcium interaction of conventional buffers and simulate physiological conditions in the plant cell. All experiments were perfomed in triplicate.

### Bioinformatics

Sequences were gathered from the annotated ORFs of the *Z. marina* genome^1^ and ZM genbank identifiers are used throughout. Sequence gathering of classical AGPs was performed using the MAAB pipeline^29^ as implemented in the [R] language (ragp package, GitHub). Chimeric AGPs were identified by first screening the genome for sequences with predicted AG regions (ragp package, GitHub) with an AG region defined by ≥3 matches to the motif “[STA]P” within a 10 residue window. This was followed by filtering for those containing at least one detectable protein domain (Pfam), and a signal peptide (SignalP 5.0). PFAM domains were annotated both by family and by clan (equivalent to superfamily). Domain structure was visualised using custom [R] scripts. Unaligned sequences for classical and chimeric AGPs are included as Supplementary Data files S1 and S2.

Glycosyltransferase (GT) enzymes from the GT14, GT31, GT61 and GT 77 families and methytransferase enzymes from the DUF579 family were identified by searching the ORFs of the *Z. marina* genome by BLAST. For each enzyme family, all *A. thaliana* sequences of the respecitive family were used as queries and duplicate returned sequences removed. For each enzyme family, sequences were aligned using Clustal Omega, and prepared for phylogetic analysis by trimming to the GT domain and applying the TrimAl-gappyout algorithm (which removes columns predicted to be phylogenetically uninformative^69^). The best fit substitution model was determined to be using Model J and a 1000 bootstrap maximum likelihood phylogeny were calculated with RAxML^70^. Aligned sequence sets for all enzyme families discussed are included as Supplementary Data S3.

## Supplementary information

Supplementary information.
