# Supplementary material for: Arabinogalactan-proteins of Zostera marina L. contain unique glycan structures and provide insight into adaption processes to saline environments
Source: Sci Rep. 2020 May 19;10:8232. doi: 10.1038/s41598-020-65135-5 (PMC7237498; doi:10.1038/s41598-020-65135-5)
Supplement: Supplementary file 1 — Supplementary information. [file 41598_2020_65135_MOESM1_ESM.pdf]

Supplementary information

Arabinogalactan-proteins of *Zostera marina* L. contain unique glycan structures and provide insight into adaption processes to saline environments

Lukas Pfeifer[1], Thomas Shafee[2], Kim L. Johnson[2,3], Antony Bacic[2,3] and Birgit Classen[1*]

Affiliations:

[1] Pharmaceutical Institute, Department of Pharmaceutical Biology, Christian-Albrechts-University of Kiel, Gutenbergstr. 76, 24118 Kiel, Germany

[2] La Trobe Institute for Agriculture & Food, Department of Animal, Plant and Soil Sciences, La Trobe University, Melbourne, Victoria 3086, Australia

[3] Sino-Australia Plant Cell Wall Research Centre, School of Forestry and Biotechnology, Zhejiang A&F University, Hangzhou, China

Corresponding author:

Birgit Classen, Pharmaceutical Institute, Department of Pharmaceutical Biology, Christian-Albrechts-University of Kiel, Gutenbergstr. 76, 24118 Kiel, Germany

Phone: +49-431-8801130

e-mail: *bclassen@pharmazie.uni-kiel.de*

Contents

Supplementary Tables **3**

Supplementary Table S1. Tissue-specific neutral monosaccharide composition of HMFs 3
Supplementary Table S2. Tissue-specific neutral monosaccharide composition of AGPs 3
Supplementary Table S3. Mass and volume of whole plant AGPs 3

Supplementary Figures **4**

Supplementary Figure S1. Mass spectrum of the uronic acid reduced sample. 4
Supplementary Figure S2. Comparison of inferred *Z. marina* and *A. sativa* glycans 5
Supplementary Figure S3. Sequences with diagnostic motifs highlighted. 6
Supplementary Figure S4. Domain families of all chimeric AGPs. 8
Supplementary Figure S5. Most common chimeric AGP domain architectures. 9
Supplementary Figure S6. Phylogeny of GT31 family members. 10
Supplementary Figure S7. Phylogeny of GT14 family members. 11
Supplementary Figure S8. Phylogeny of arabinosyltransferases families. 12

Supplementary Data Files **13**

Supplementary Data S1. Classical HRGPs from MAAB (signalP filtered) 13
Supplementary Data S2. Chimeras by clan (signalP filtered) 13
Supplementary Data S3. Enzyme trimmed alignments and trees 13

Supplementary Tables

Supplementary Table S1. Tissue-specific neutral monosaccharide composition of HMFs

Neutral monosaccharide composition of high molecular weight fractions (HMF) from *Z. marina* in % (mol mol^{-1}).

Neutral monosaccharide	*Z. marina* whole plant (n=3)	*Z. marina* leaves (n=1)	*Z. marina* rhizome (n=1)	*Z. marina* root (n=1)
Gal	31.0 (± 0.8)	33.6	29.6	30.9
Ara	29.5 (± 1.7)	28.9	30.7	18.6
Rha	7.1 (± 0.1)	2.3	8.5	13.0
Man	6.8 (± 0.5)	12.0	4.9	6.5
Glc	12.5 (± 0.6)	14.2	17.1	13.5
Xyl	11.7 (± 0.8)	9.0	9.2	15.8
Fuc	1.4 (± 0.8)	-	-	1.7

Supplementary Table S2. Tissue-specific neutral monosaccharide composition of AGPs

Neutral monosaccharide composition of AGPs from the different organs of *Z. marina* in % (mol mol^{-1}).

Neutral monosaccharide	*Z. marina* whole plant (n=3)	*Z. marina* leaves (n=3)	*Z. marina* rhizome (n=3)	*Z. marina* root (n=3)
Gal	43.0 (± 0.4)	49.2 (± 0.8)	41.1 (± 4.2)	48.6 (± 0.9)
Ara	41.1 (± 0.5)	38.8 (± 1.0)	38.9 (± 2.1)	32.9 (± 0.7)
Rha	6.1 (± 0.0)	2.3 (± 0.1)	8.8 (± 1.1)	9.0 (± 1.0)
Man	4.6 (± 0.3)	3.8 (± 0.5)	3.6 (± 0.3)	2.1 (± 0.8)
Glc	3.0 (± 0.7)	4.7 (± 1.0)	7.1 (± 1.9)	6.4 (± 0.9)
Xyl	2.2 (± 0.5)	1.2 (± 0.1)	0.5 (± 0.3)	1.0 (± 0.4)

Supplementary Table S3. Mass and volume of whole plant AGPs

Determination of absolute molecular weights and hydrodynamic volumes of different whole plant AGPs from *Z. marina* (kDa)

Sample	Absolute molecular mass (main peak*)	Hydrodynamic volume (main peak*)
Z. marina	240.0 (± 0.3 %)	47.0
Z. marina UR	165.2 (± 0.2 %)	40.7
Z. marina Ox	164.1 (± 0.4 %)	12.8

*main peak has a minimum mass recovery of 30% (m m^{-1})

UR, uronic acid reduced; Ox, oxalic acid hydrolysed.

Supplementary Figures

Supplementary Figure S1. Mass spectrum of the uronic acid reduced sample.
Mass spectrum and fragmentation pattern of C6-di-deuterated 4-OMe Glc in the uronic acid reduced sample of *Z. marina* whole plant AGP after alditol acetate analysis. In green the origins of the five most intense fragments, aside from the acetyl-group (m/z = 43.0), are indicated.

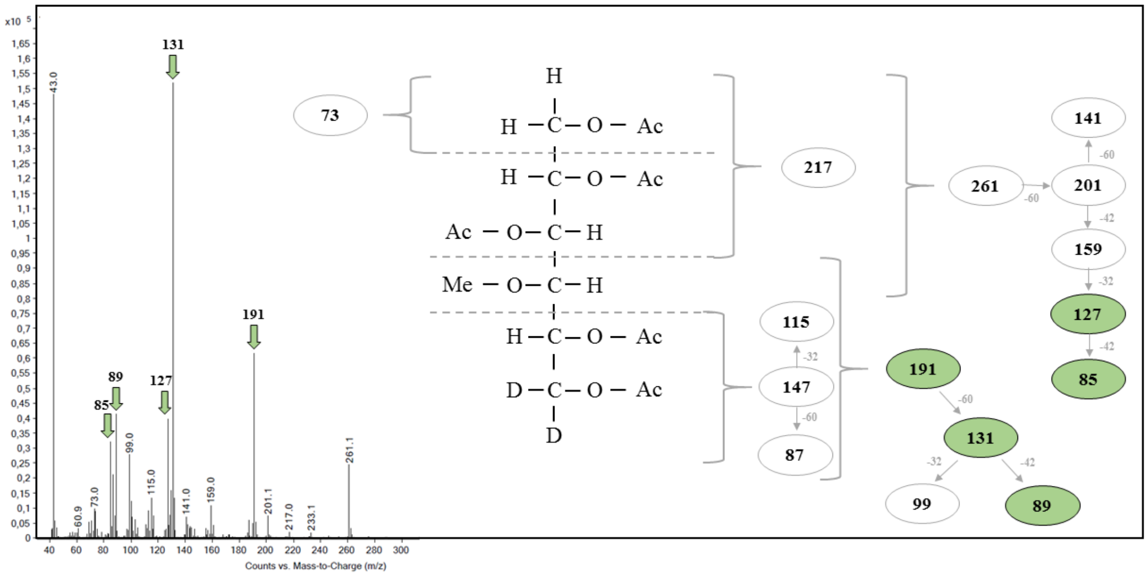

Supplementary Figure S2. Comparison of inferred *Z. marina* and *A. sativa* glycans. Comparison of **a** *Z. marina* AGP (as presented in the main text) and **b** *A. sativa* AGP based on linkage-type analysis results from [25].

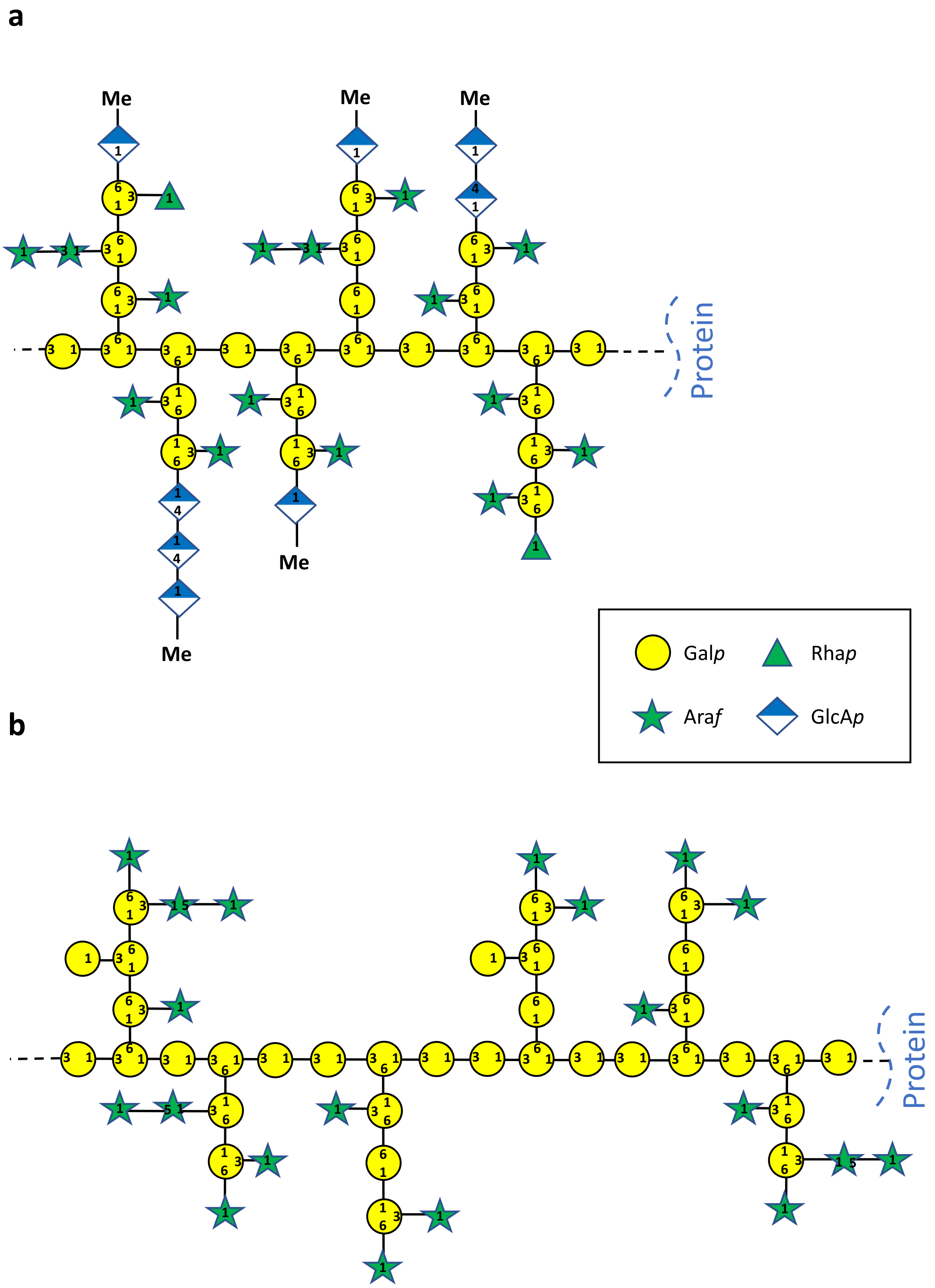

Supplementary Figure S3. Sequences with diagnostic motifs highlighted.

Sequences identified by MAAB in *Z. marina* with diagnostic motifs highlighted and shown by MAAB class [30]. Signal peptide in green, GPI anchor in purple and motifs used for classification of sequences are AGP motifs ([ASVTG]P, [ASVTG]PP, [AVTG]PPP) in cyan, Extensin motifs (SP3, SP4, SP5) in red and cross-linking motifs ([FY]XY, KHY, VY[HKDE], VxY, YY) in dark blue and PRP motifs (PPV[QK], PPVx[KT] and KKPCPP) highlighted brown. The total number of sequences identified in each class is shown in brackets. * indicates putative alternative start site.

Class 1: GPI-AGPs (5)

>KMZ66553.1 hypothetical protein ZOSMA_294G00050
MAIIPRYLQHLAVFFVFISVLFATTLAQALTDQSPLASTQPPSTSQLSPTFSASPTPTPTSSPSATTPASLPP
TPSPSAASTPTISPTPSPSPSAVSTSTIAPTLPPLAASPLSDGPSGLSSPAESPRSSGSSSLVGCTGVGFAAL
VLTLL

>KMZ65764.1 hypothetical protein ZOSMA_30G00450
MAITRTAISLFSIFLLASTSIFAQSPATAPTLPPPAPVASTPPPISVSSPPSVVATPPPVSAAPPMMVSTPPM
SAPVPMAAEVPSVETPVDSPVADTPDEAMVPEASMPASPVPSSFAPNIPNPEGSAGSTGAKMNVVGLMGLVGA
AAMLI

>KMZ63928.1 hypothetical protein ZOSMA_38G00360
MTHRSILTVVFLLSIIASLHDAAVFTSAADSPTTSPSPASPPPTATATAPTTTTTTSVPPPTATPPTTNVTAV
QPPTATPPTTNVTAVQPPTATAPTTSSITTPAASPTTTAAPATPPTTNVTPISAPVSSPPTPPTPAPTTITPA
PSLPVEVPAPAPTKSKKKPSLPLAPSPSPDSINLAPSSEAPGSISDDFLAADTADAATGGNGVVGFGLLVVSL
LVAVV

>KMZ59493.1 hypothetical protein ZOSMA_68G00800
MGIRIQVTLIAFAILFATVIAQQAPASAPTSTPGSVSSPPSVSSPSSLSSPPSISSPPSLSSAPSSSPPSMAP
PSDQAPSAPMTPEALASSGSPGSAPSISQENNAASFAASWIGAAGSVALVMAYAF

>KMZ56883.1 hypothetical protein ZOSMA_8G00260
MAAAPTFSFSAMLLHLLFLFLSLSVFSQAKMASPISAISAAPAFLPEAPSSLLSPYPSYSPTLSPDGSMQPEF
PTPRAEGVAPTTSIITSVQSPPNPDTMVPEAGDDDGFFVLAPVGYSASIAADTSSASSRVVAIRLIVVFLVLK
SSLILLLVSTFSC

Class 4: non-GPI-AGPs (4)

>KMZ70582.1 hypothetical protein ZOSMA_199G00190
MASHNLLWLLIIACFCSSLSTTTYAQSQSASPVTLPVAPVIPVSSPPPPKTLASTPTTSSPLASPVIPVSSPP
PPKTLASTPTSSPLASPTLPVAPVLPVSSPPPPKILASTPTPPPSSPSPIIQTPSPPPVLSLPPVSLPPALPI
TPAPAALAITPAVSPSPAADSPLAETPALAPAPVHPKHKHKHRRWHRRKHRRHKKHHVMAPAPAPIPPSPPSP
PAPPEFDDYTIDGPSPAPTDLSGGTSIHGIYKHGRRLSEGLRLTNFILIPFLLFLF

>KMZ62892.1 hypothetical protein ZOSMA_43G00740
MGDLRSTTAAVLLILYFNMSSAARILEGGGGGGGGGGGGGGGGNGGYGYGFGRGAGYGYGYSAPGAPTSSSPI
YSQNSTTNQGSCSAPNTPSPPPPPKEPCAPNTTVTPPPKEPCTPNTTVTPPPKEPCAPNTTVTPPPKEPCAPN
TTVTPPPPPPSPPVISPGPPSPSPPVISPGPPPPPVISPPTPVETPPPPPNSVDTPPPPPTPVETPPPPPNSV
DTPPPPPTPVETPPPPPNSVDTPPPPPAPVETPPPPPNSVETPPPPPNSVDMPPPPPNSVDMPPPPPTPVETP
PPPPTPVETFPPPPPLNLTPPPPSLLDDEPPPLPPLVETPPPPVTPSTPTTTLPLIGVLSPKEPCTPNTPSL
SPPPPKEPCSPNTPSLSPPPPPPPPPEEPCTPNTPSLSPPPKEPCAPNTPSLSPPSHTKIPLQPFPKTPSFPH
NENGKNWGFGSGSGFGHGGAFGGTYGSFGGGGGGGGGGGGGGGGNDASGYGSGYGNGMGYGSSFGGNDFMNAG
GGGGGGGGGGGFAGMGNYDYRQFGGGGGGGGGGGGSGGFGQFGGGGGGGGGGGGGGGGF

>KMZ58528.1 hypothetical protein ZOSMA_76G00830, partial
MKQKWELAWFLVACDVLLFSTLMDFPIVCGGSKGKALSPAPKVISSTLTEPTVPSISPLPSLLPSPPLFSTVP
MIPNVSPPISRLHMPPVPIAPTIPNVSPSSLPLISPSLVPIVSEIPNVSPPSLPQFSPSLVPMVSEIPNVSHS
SLLPSPPPTPITSTISNVSPSSLLPPSPSLLPMTPITSNISPPLLHPPSPLLPIPAKILNSSPPQLSLFSPPP
ASTASTAPNFSPSSLLPHSPLLPIPVTKPNLSHPPLPLLNPPLALTTSTPPKVSPPLLPLPSPPVTIAPTVHN
VSTPFLPHNPSHFLKQPPVQNISTPPLLSSPPPVLIVPPLHNISAPLFPSPPLAHIAPTIENIPSLVPPSFPL
GRSPSQQDRSSSAPIDRHPRRTLDSPPARPKNSIPTQSSPSHSPSLAPDASTSAGTASHLSHNHHSPVRGSVP
VSSPKPPKSLTYSPLGSHTRFPNRSKLLHSSSAAPNSHHHQSRNTSSISPSNDK

>KMZ57387.1 hypothetical protein ZOSMA_86G00220
MDHRSVATVGLLMCIVAMSVGVHAMSPASASAPTTITSEISPTSESSPVADTPSSPTVIESPPTPAPVVDAIP
DSPPSMEVNSPPTPAPVVDTIPPTVSIPDSPPPMEVNSPPTPPPVVDAIPPTVSIPDSPPPMEINSPPTPAPV
VVTSSPDSPPPVENSPPTPAPVVNAAPPPMSSLPDSPPPVENSPPTPAPVVDAAPPPMSSLPDSPPPVENSPP
TPAPIVAPATPPPITSLPDTPPPVVASVPRSGAPVVASAPRSVLVPTSAPLSPPTPAASPPTTILSSPVPAPV
EVTPDGSPFVDAPAPSSTSVAPSLSEAFPPGPSPDPAIFADDTARGVKENPMIAGAVITMLMLMSSFAVVFF

Class 2: CL-EXT (1)

>KMZ72364.1 hypothetical protein ZOSMA_166G00640
MTEGRDGPERGRRRRKFLPSI*MVAAFAVVLVVTTNVGVVSGDPYIYSSPPPPYNYESPPPPPYKYESPPPPP
YKYESPPPPPYKYESPPPPPYKYKSPPPPPYKYESPPPPPYKYESPPPPPYKYESPPPPPYKYESPPPPPYKY
ESPPPPPYKYESPPPPPYKYESLPPPPYKYESPPPPPYKYESPPPPPYKYESPPPPPYKYESPPPPPYKYKSP
PPPPYKYESPPPPPYKYESPPPPPYKYKSPPPPPYKYESPPPPPYKYESPPPPPYKYESPPPPPYKYKSPPPP
VYKYESPPPPPYKYKSPPPPAYKYESPPPPPYKHESPPSYYHKSPPPKPYYPPHHNRIFKVVGEVYCYGCYDS
EHSPKSHHKKKLEGAVVKVTCMKGSKDVIAYGKTMRNGMYEITVEDYDVDKYGHENCKAMLHAPPKGSSCNMA
TDIHNGNTGAKLHIKSRNHVEVVYKSKKFAYAPKTPYKDCYEKRHHHHHHHHSPVPYCKPPPPTYYYKSPPP
PTPTYYYKSPPPPAPTYHYKPPSPKYYYKSPPPPSPKYHYKSPPPPSPKYHYKSPPPPSPKYYYKSPPPPAPT
YYYKPPSPKYYYKSPPPPSPKYYYKSPPPPVHSPPPPYYYKSPPPPVHSPPPPYYYKSPPPPVHSPPPPYYYK
SPPPPEHSPPPPYHYKSPPPPEKSPPPPYHYESPPPPEKSEPHPYIYSSPPPPIYK

Class 20: Shared Bias, high EXT (SPn & Y) (1)

>KMZ74158.1 hypothetical protein ZOSMA_133G00070
MRKITASFFLILTLAALASPGDSADSAKLIGVAECADCGNNAFGSFKGINVAVVCNSEINLVDFKEVAVGEFA
GDGKLSLQLPTTIVDKKCFAHVRSLSKTNPCPTFQNLDNFILSLSSDDQSVYVFGNSDGKVSFSRAACAQKTF
WKYPYFKCPNHPWFKYLPYCNPPPSNPPPVYKNPPPVYKNPPPVYKNPPPVYKNPPPVYKNPPPCNPPPYKKP
VYKSPPPVHKSPPPVHKNPPPCNPPPYKKPVDKNPSPYKPPVHNTPPMHMKPPSPKYNPPVHKPAPCNTPIHK
PPMFKLPPIYKPPVYVPKHPKTTTSN

Class 21 Shared Bias, high SPn (1)

>KMZ66204.1 hypothetical protein ZOSMA_2G02140
KPLFVLVLILLLFAIVSTVKADERRRCVRGCHRRLHRCRNTTYSHGHRHCLRKYRRCIIICNNKLPPPPPPPP
SPPPPSPLPSCAHCVRGCYQRLHRCRNTTYHHGHRHCLRRYRRCIIICNNKFPPPPPSPPPPSPPPPPPPPPS
PPPPSPPPPSPPPPSPPPPPPPPSPPPPSPPPPPPPPSPPPPPSPPPPPPPSPPPPSPPPPPPSLPPSSPPPP
PPPPLCFTTLKSCVNGCGHNDPCVLKCSNDYKVCRSNVPIIDHTK

Class 24: <15% motif HRGP (3)

>KMZ58455.1 hypothetical protein ZOSMA_76G00110
MKPCVVSLFVIAAISLQIILVAPLVCGRTLQDFDDQKAYYHNSPPKTSHSGSHSSHSKGSSFPSPPHQGG
CAKTPSHSSSSSTPKPRDGSYGTPTTPSHGSRSATTPATTPPTSSHATTPTTPSHSTTPSTPSHTTTPAT
PSIPGFPSITATCDFWRTHPSMIFGILGQWSNIGNLFGFPATSIFGRNPSVPQALGNARNDGYGALFREG
TASLLNSMANPSFPLTTQVVRDRFNQALSSEKTASAEAQRFLANEGA

Supplementary Figure S4. Domain families of all chimeric AGPs.

Pfam domains found in sequences containing at least 1 AG region and a predicted signal peptide. For those sequences with multiple Pfam domains, only the largest domain is counted.

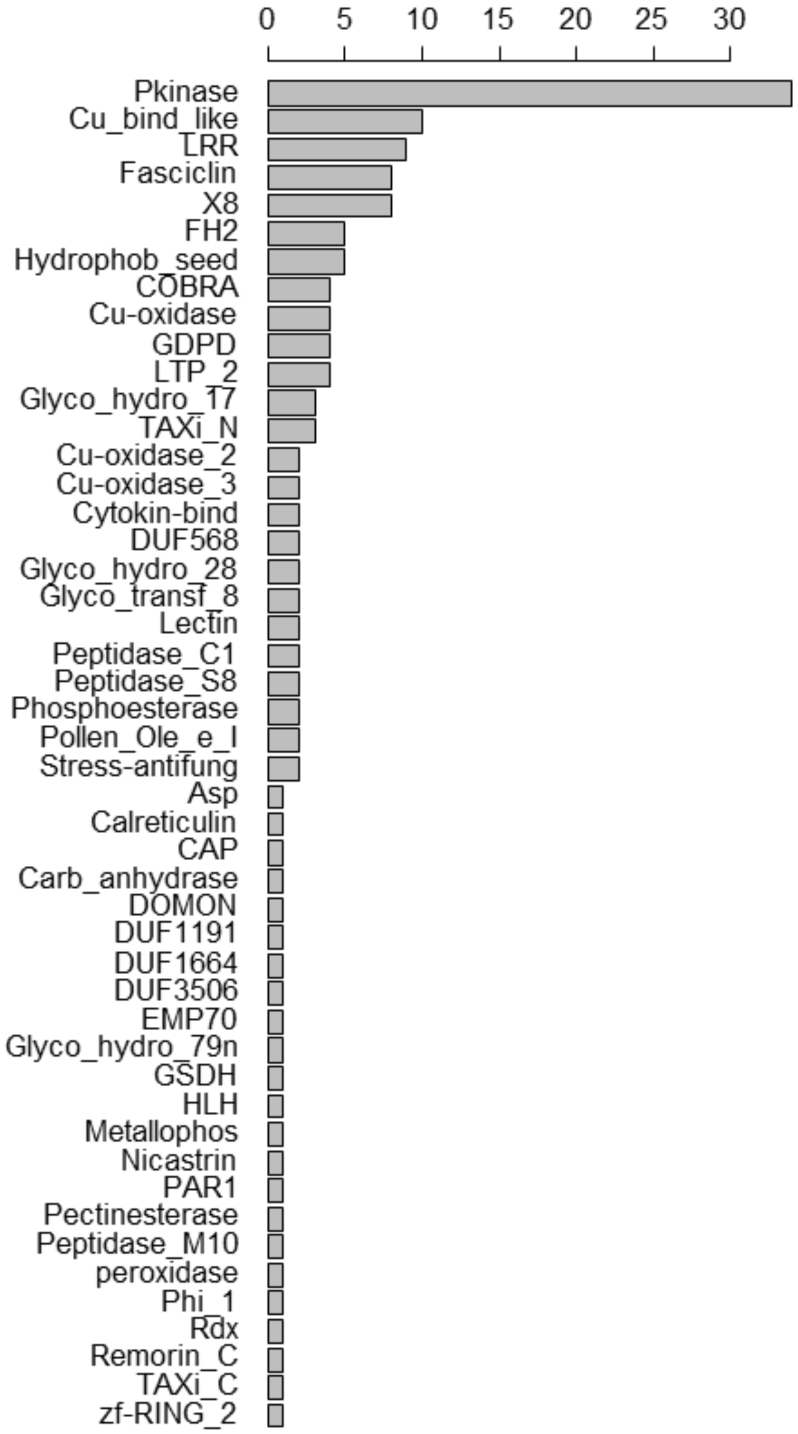

Supplementary Figure S5. Most common chimeric AGP domain architectures. Architectures in *Z. marina* with domains coloured by their Pfam family (see Fig. 5b). AG regions in white.

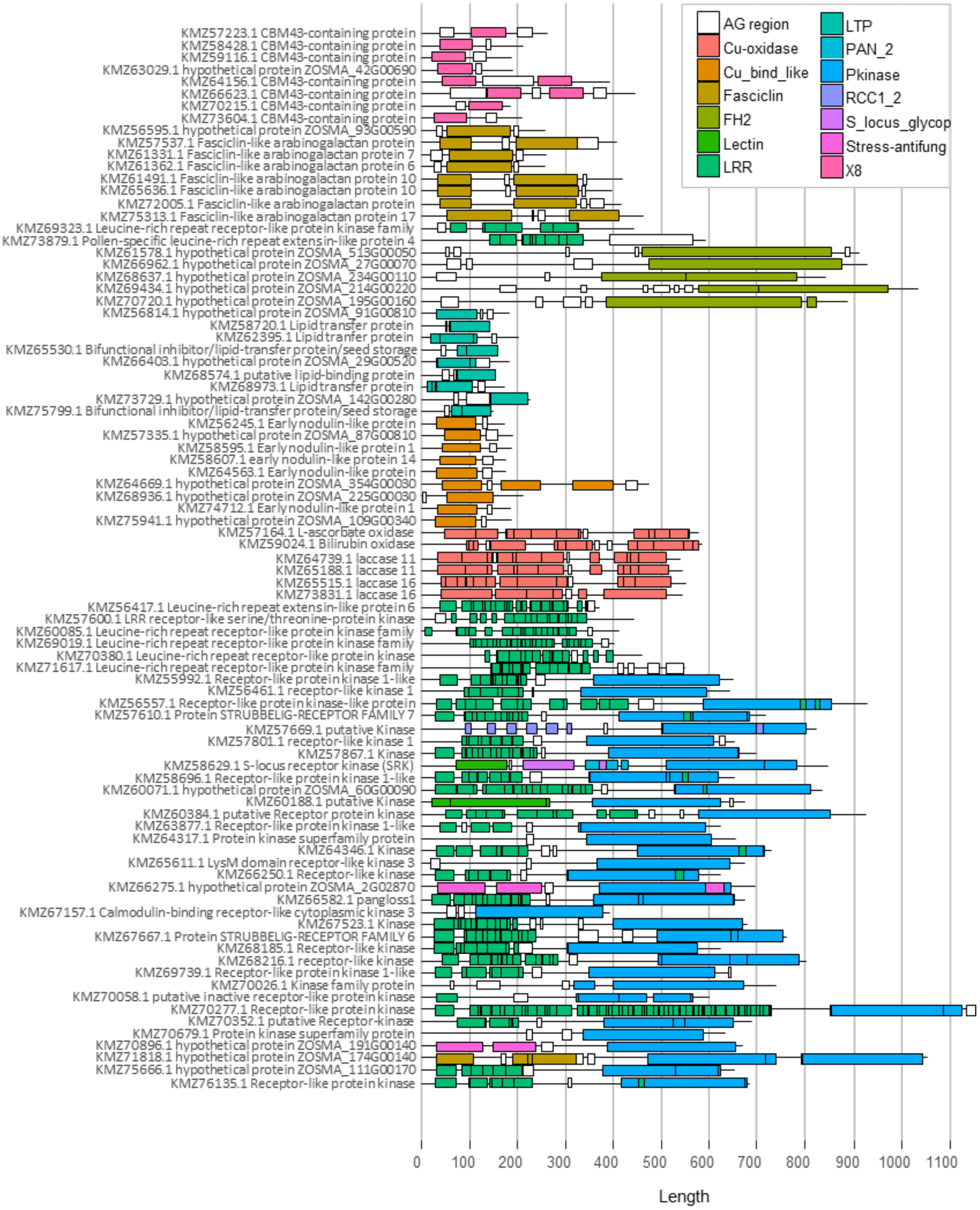

Supplementary Figure S6. Phylogeny of GT31 family members.

Members from *Z. marina* (ZM accession numbers, highlighted with black circles), *Arabidopsis thaliana* (AT). Clades labelled as in [31]. Genes with known function indicated. * Note: although QMAP8 is described as a β-1,6 GalT in the published literature, unpublished data suggests that it may be a β-1,3 GalT.

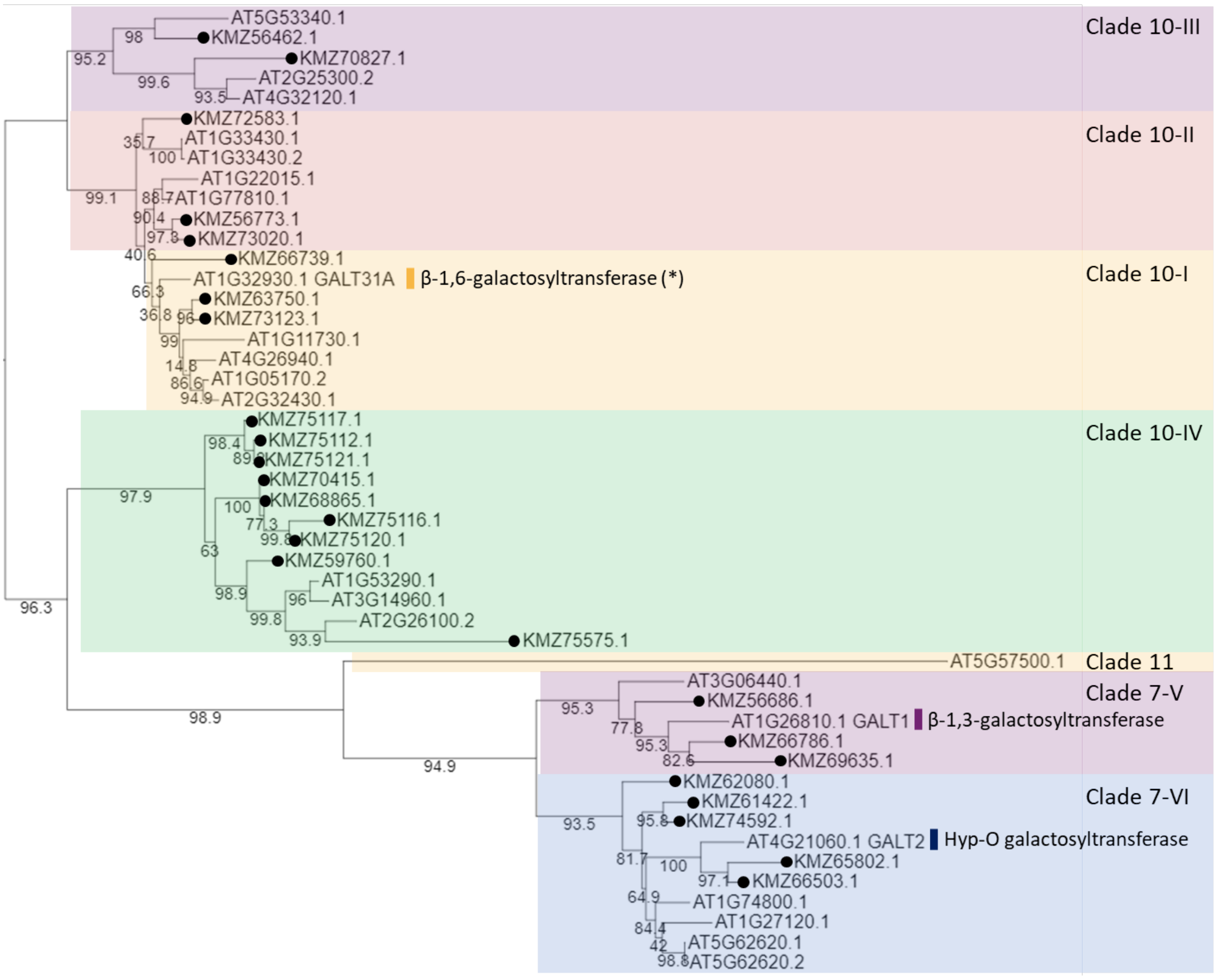

Supplementary Figure S7. Phylogeny of GT14 family members.

Members from *Z. marina* (ZM accession numbers, highlighted with black circles), *Arabidopsis thaliana* (AT). Clades labelled as in [32]. Genes with known function indicated.

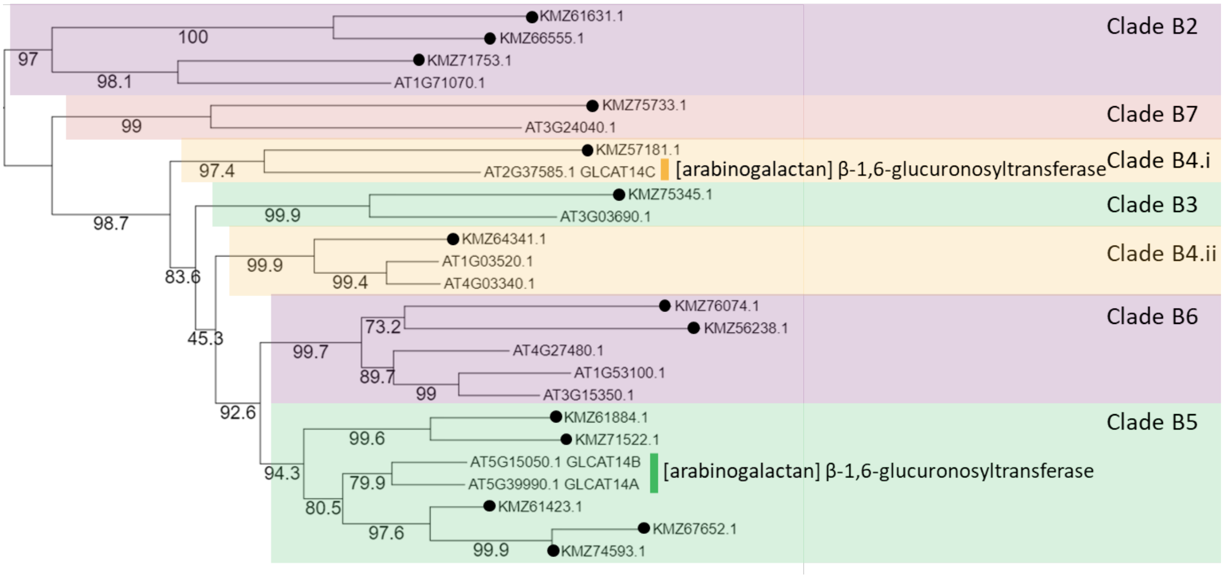

Supplementary Figure S8. Phylogeny of arabinosyltransferases families.
a Phylogeny of GT61 family members from *Z. marina* (ZM accession numbers, highlighted with black circles), *Arabidopsis thaliana* (AT). Clades labelled as in [33]. Genes with known function indicated. **b** Phylogeny of GT77 family members from *Z. marina* (ZM accession numbers, highlighted with black circles), *Arabidopsis thaliana* (AT). Clades labelled as in [36]. Genes with known function indicated.

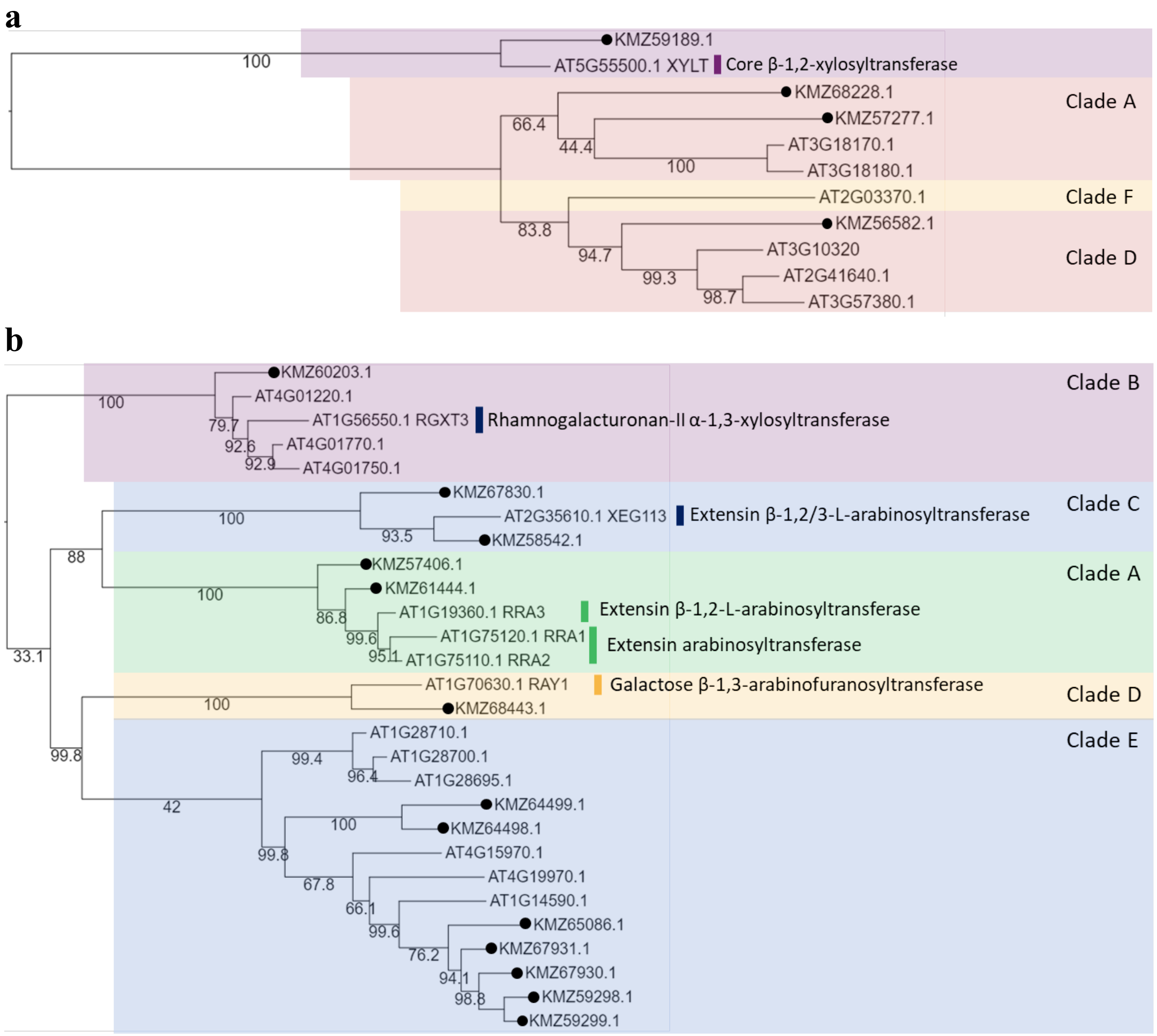

Supplementary Data Files

Deposited at DOI: /10.26181/5e8c491575d77

Supplementary Data S1. Classical HRGPs from MAAB (signalP filtered)
Unaligned sequences of HRGPs as fasta file

Supplementary Data S2. Chimeras by clan (signalP filtered)
Unaligned sequences of chimeric AGPs as fasta files, organised by clan of the globular domain

Supplementary Data S3. Enzyme trimmed alignments and trees
Multiple sequence alignments of enzymes as fasta files and phylogenetic trees as newick files
